# KPT6566 induces apoptotic cell death and suppresses the tumorigenicity of testicular germ cell tumors

**DOI:** 10.3389/fcell.2023.1220179

**Published:** 2023-11-02

**Authors:** Ruijing Sun, Eun Joo Lee, Seonock Lee, Gamin Kim, Jungho Kim

**Affiliations:** Laboratory of Molecular and Cellular Biology, Department of Life Science, Sogang University, Seoul, Republic of Korea

**Keywords:** KPT6566, P19, NCCIT, testicular germ cell tumor, Oct-4, Sox2

## Abstract

Testicular germ cell tumors (TGCTs) frequently affect adolescent and young adult males. Although TGCT is more responsive to cisplatin-based chemotherapy than other solid tumors, some patients are nonresponders, and following treatment, many patients continue to experience acute and long-term cytotoxic effects from cisplatin-based chemotherapy. Consequently, it is imperative to develop new therapeutic modalities for treatment-resistant TGCTs. Peptidyl-prolyl isomerase (Pin1) regulates the activity and stability of many cancer-associated target proteins. Prior findings suggest that Pin1 contributes to the pathogenesis of multiple human cancers. However, the specific function of Pin1 in TGCTs has not yet been elucidated. TGCT cell proliferation and viability were examined using cell cycle analysis and apoptosis assays following treatment with KPT6566, a potent, selective Pin1 inhibitor that covalently binds to the catalytic domain of Pin1. A xenograft mouse model was used to assess the effect of KPT6566 on tumor growth *in vivo*. KPT6566 effectively suppressed cell proliferation, colony formation, and ATP production in P19 and NCCIT cells. Further, KPT6566 induced apoptotic cell death by generating cellular reactive oxygen species and downregulating the embryonic transcription factors Oct-4 and Sox2. Finally, KPT6566 treatment significantly reduced tumor volume and mass in P19 cell xenografts. The Pin1 inhibitor KPT6566 has significant antiproliferative and antitumor effects in TGCT cells. These findings suggest that Pin1 inhibitors could be considered as a potential therapeutic approach for TGCTs.

## Introduction

Human testicular germ cell tumors (TGCTs) are a prevalent cancer type that primarily affects adolescent and young adult males (Andrews, 1988; Horwich et al., 2006; Campanner et al., 2023). Recent findings have identified a persistent increase in TGCT incidence over the past decades, and these malignancies are now the most common cause of cancer-related mortality and morbidity in this age group (Batool et al., 2019; Al-Obaidy et al., 2020; Miller et al., 2020; Siegel et al., 2020; Siegel et al., 2020). Germ cells, which are responsible for sperm production, are the predominant origin of testicular cancer. Based on histological features, prognostic implications, and treatment approaches, TGCTs are classified into two categories: seminomas and nonseminomas (Winter and Albers, 2011). The traits of seminomas are consistent with primitive germ cells, while nonseminomas are most commonly comprised of embryonal carcinoma cells. Embryonal carcinoma, a nonseminoma subclass, originates in the testicles and is characterized by rapid proliferation and metastasis. These carcinomas have pluripotent properties and express pluripotency markers, including the Oct-4 and Nanog embryonic transcription factors (de Jong et al., 2005; Hart et al., 2005). Although cisplatin-based chemotherapy is more effective against TGCTs than other solid tumor types, some patients are nonresponders, and many patients experience acute and chronic cytotoxic effects (Singh et al., 2019). Consequently, developing novel, efficient therapeutic strategies targeting refractory TGCTs is urgently needed to supplement traditional chemotherapeutic approaches.

Peptidyl-prolyl *cis/trans* isomerase or NIMA-interacting-1 (Pin1) plays a crucial role in development of many human cancers (Lu and Hunter, 2014; Zhang et al., 2020; Winter and Albers, 2023). Pin1 is commonly overexpressed or hyperactivated in diverse human cancer types, and its expression is commonly associated with poor patient prognoses (Wulf et al., 2001; Bao et al., 2004; Wulf et al., 2004; Lu and Hunter, 2014; Luo et al., 2014; Rustighi et al., 2014). Pin1 is a peptidyl-prolyl cis/trans isomerase that targets phosphorylated serine-proline (pS-P) or phosphorylated threonine-proline (pT-P) motifs within target proteins (Yaffe et al., 1997). The subsequent *cis-trans*-isomerization of target proteins induces conformational alterations critical for modulating their biological function and stability (Lu et al., 2007; Makinwa et al., 2020). Several studies have documented that Pin1 regulates a myriad of proteins implicated in cancer progression, including 1) cell cycle regulators (Cyclin D1, Cyclin D2, Cyclin D3, Cyclin E, CDK4, and CDK6), 2) oncogenic proteins (c-Jun, c-Myc, and β-catenin), and 3) tumor suppressors (p53, p63, and p73) (Ryo et al., 2001; Wulf et al., 2001; Liou et al., 2002; Zacchi et al., 2002; Zheng et al., 2002; Yeh et al., 2004; Zhou and Lu, 2016; Kim et al., 2022). Pin1 hyperactivation promotes tumorigenesis and negatively affects clinical outcomes (Wulf et al., 2001; Wulf et al., 2004; Lu and Hunter, 2014; Luo et al., 2014; Rustighi et al., 2014). The tumoricidal effects of small-molecule Pin1 inhibitors suggest Pin1 plays a pivotal role in tumor development (Kim et al., 2009; Wei et al., 2015; Campaner et al., 2017). Our recent work demonstrated that small-molecule Pin1 inhibitors have antitumorigenic activity against colorectal cancer stem cells or tumor-initiating cells (Kim et al., 2022). KPT6566 {2-[(4-(4-*tert*-butylbenzenesulfonamido)-1-oxo-1,4-dihydronaphthalen-2-yl)sulfanyl]acetic acid}, a potent Pin1 inhibitor, was identified by inhibitor screening of a chemical library (Campaner et al., 2017). KPT6566 selectively inhibits the peptidyl-prolyl cis/trans isomerase activity of Pin1 by covalently binding its catalytic domain.

Octamer-binding transcription factor-4 (Oct-4), also known as POU domain, class 5, transcription factor (POU5F1), is an embryonic transcription factor predominantly expressed in pluripotent cells of developing embryos and is crucial for maintenance of pluripotency (Nichols et al., 1998). Prior findings robustly support a pivotal role for Oct-4 in human TGCTs (Gidekel et al., 2003; Jones et al., 2004; Ezeh et al., 2005; Richie, 2005; Cheng et al., 2007). Additionally, Oct-4 is expressed in many human cancers, including lung cancer (Chen et al., 2008), breast cancer (Ezeh et al., 2005), liver cancer (Huang et al., 2010; Wang et al., 2010), gastric cancer (Chen et al., 2009), and bladder cancer (Atlasi et al., 2007). Intriguingly, emerging findings have identified a role for Oct-4 in maintaining cancer stem cell or tumor-initiating cell characteristics (Hu et al., 2008) and development of chemoresistance in tumor cells (Linn et al., 2010; Wang et al., 2010). Moreover, ablation or inhibition of Oct-4 hinders proliferation and augments chemotherapy efficacy in cancer stem-like cells (Chen et al., 2008; Hu et al., 2008; Yun et al., 2015). Sox2 is an additional embryonic transcription factor in the Sex-determining region of the Y chromosome (Sry)-related high-mobility group (HMG) box gene family (Kamachi et al., 2000; Boyer et al., 2005; Episkopou, 2005). Sox2 is essential for maintenance of embryonic stem cells (Avilion et al., 2003) and induction of pluripotency (Avilion et al., 2003; Takahashi and Yamanaka, 2006; Takahashi et al., 2007). Recent findings have associated aberrant Sox2 expression with tumorigenesis in diverse cancers, including bladder cancer (Amini et al., 2014; Zhu et al., 2017), breast cancer (Rodriguez-Pinilla et al., 2007), colorectal cancer (Han et al., 2012; Amini et al., 2014), gastric cancer (Li et al., 2004), glioma (Alonso et al., 2011; Fang et al., 2011), osteosarcoma (Basu-Roy et al., 2012), pancreatic cancer (Sanada et al., 2006), prostate cancer (Amini et al., 2014), and skin squamous cell carcinoma (Boumahdi et al., 2014). Further, Sox2 is activated in cancer stem cells or tumor-initiating cells (Leis et al., 2012). Sox2 expression is associated with maintenance of cancer stem cells or tumor-initiating cell proliferation (Basu-Roy et al., 2012), regulation of the epithelial-to-mesenchymal transition (EMT), and increased tumor cell migration and invasion (Han et al., 2012). These findings indicate that Oct-4 and Sox2 function as oncogenic transcription factors, and that Oct-4 and Sox2 expression contribute to the development and progression of various human cancers.

Prior studies have demonstrated that Pin1 is integral to the development of various human malignancies, but its role in TGCTs remains unknown. The purpose of the present study was to elucidate the effects of Pin1 and KPT6566 on TGCT carcinogenicity using P19 and NCCIT embryonal carcinoma cell lines. These findings demonstrated that KPT6566 inhibited cell proliferation and colony formation, induced apoptosis, and downregulated expression of Oct-4 and Sox2 in embryonal carcinoma cells. Furthermore, KPT6566 significantly decreased the tumorigenic capacity of P19 embryonal carcinoma cells *in vivo*. Together, these findings suggest that targeting Pin1 with small-molecule inhibitors is potentially an effective therapeutic strategy for TGCTs.

## Materials and methods

### Cell culture

P19 and NCCIT cell lines were procured from the American Type Culture Collection (ATCC) and cultivated in Dulbecco’s Modified Eagle’s Medium (DMEM; ThermoFisher Scientific) supplemented with 10% heat-inactivated fetal bovine serum (FBS; Sigma-Aldrich), GlutaMAX (ThermoFisher Scientific), and 1% Penicillin-Streptomycin (ThermoFisher Scientific). Cells were maintained in a humidified 37°C incubator (ThermoFisher Scientific) with 5% CO_2_. Pin1 knockout (Pin1^−/−^) mice were kindly provided by Dr. Kun Ping Lu (Western University, Canada), and mouse embryonic fibroblasts from wild-type and Pin1^−/−^ mice were isolated from E13.5 embryos as described previously (Oh et al., 2016). Procedures used to generate mouse embryonic fibroblasts were conducted in strict accordance with the guidelines for animal experimentation established by Sogang University. Prior to beginning the study, all protocols were approved by the institutional animal care and use committee (IACUCSGU 2022_09).

### Cell growth curve

Duplicate samples of 2 × 10^4^ P19 or NCCIT cells were plated in 12-well plates and cultured for 5 days. Cells were observed daily using an inverted phase-contrast microscope (IX71; Olympus). Total cell count was recorded at 1 day intervals for 5 days using a hemocytometer.

### Cell counting Kit-8 (CCK-8) assay

Cell viability assays were conducted according to the manufacturer’s instructions using a Cell Counting Kit-8 (Sigma-Aldrich). P19 or NCCIT cells (2 × 10^3^) were seeded into a 96-well plate and grown in the presence of varying concentrations of KPT6566 (CSNpharm) for 5 days. Subsequently, the cells were combined with 10 μL CCK-8 solution/well and incubated for an additional 3 h at 37°C. Formazan dye produced by cellular dehydrogenase activity was quantified by measuring absorbance at 450 nm with a microplate reader (Molecular Devices). The optical density values of each well were used to determine the viability of P19 or NCCIT cells.

### Colony formation assay

The colony formation assay was conducted by seeding 2.5 × 10^3^ P19 or NCCIT cells in 12-well plates and subsequently exposing the cells to various concentrations of KPT6566. After 5 days, the colonies were fixed with a solution containing 0.05% (w/v) Crystal Violet (Sigma), 1% formaldehyde (Sigma), 1% methanol (Sigma), and 1 × PBS for 20 min. Colonies were then washed with tap water and photographed after water removal. To measure the formation of P19 or NCCIT cell colonies after treatment with KPT6566, ImageJ (https://imagej.nih.gov/ij/) was used to count the colonies stained with Crystal Violet, and colony numbers were compared to those of the untreated control groups.

### ATP assay

ATP production was measured using an ATP Assay Kit (Abcam). P19 or NCCIT cells were collected by trypsinization, washed with PBS, and subsequently combined with 100 μL ATP assay buffer. Cells were then lysed and centrifuged to remove solid particles. The supernatant was collected and mixed with an ATP probe. Absorbance was measured at 570 nm using a microplate reader (Molecular Devices). Experimental findings were expressed as percent ATP production relative to the control group.

### Determination of IC_50_ values

Two thousand P19 or NCCIT cells were seeded into 12-well plates, and KPT6566 was added to cell culture medium in varying concentrations, ranging 0–40 μM. After 5 days, viable cell numbers were measured using an automatic cell counter (ADAM MC Auto Cell Counter, NanoEnTek Inc.). Average P19 or NCCIT cell numbers were plotted against KPT6566 concentration in a sigmoidal curve using SOFTMAX PRO software (Molecular Devices). The concentration of KPT6566 that induced 50% of the maximal inhibition was reported as the IC_50_.

### Cell cycle analysis

After KPT6566 treatment, P19 and NCCIT cells were collected and fixed in 70% ethanol for 24 h at 4°C. Fixed cells were then washed twice with PBS, reactivated in PBS containing 100 μg/mL RNase A, and incubated at 37°C for 30 min. Cells were then incubated with propidium iodide (PI, 33 μg/mL) containing 10% NP-40 for an additional 30 min and subjected to flow cytometry analysis with a FACSCalibur (BD Biosciences).

### Apoptosis assay

Measurement of apoptosis was conducted using the instructions provided by the manufacturer of the FITC-Annexin V Apoptosis Detection Kit with PI (BioLegend). Flow cytometry was used to assess P19 or NCCIT cells after exposure to KPT6566. Cells were first washed twice with cold Cell Staining Buffer (BioLegend) and subsequently suspended in Annexin V Binding Buffer (BioLegend). FITC-Annexin V and PI were used to stain cells, which were then analyzed using a FACSCalibur flow cytometer.

### Western blotting

Cell extracts were separated by SDS-PAGE, transferred to PVDF membrane, and subsequently probed with primary antibodies, including anti-Oct-4 (Santa Cruz Biotechnology), anti-Sox2 (Santa Cruz Biotechnology), anti-β-catenin (BD Transduction Laboratories), anti-Cyclin D1 (Invitrogen), anti-Pin1 (Proteintech), or anti-β-Actin (AbClon). Reactive bands were then labeled using Western Lightning reagent (PerkinElmer Life Sciences) and detected by chemiluminescence.

### Xenografts

Eight-week-old male nude mice (Orient Bio Inc., Korea) were implanted on the flanks with 1 × 10^7^ P19 cells. When tumors reached a measurable size (15–25 mm^2^), mice received intraperitoneal injections of either 5 mg/kg KPT6566 or vehicle control (1% DMSO) every 3 days for 27 days. To measure tumor volume, the longest length and the greatest width were recorded using an external caliper. The modified ellipsoidal formula was used to calculate tumor volume [tumor volume = (length × width^2^)/2], as previously reported in prior studies (Euhus et al., 1986; Tomayko and Reynolds, 1989; Kim et al., 2021; Kim et al., 2022). Twenty-seven days after initiation of KPT6566 treatment, tumor-bearing mice were euthanized. Experimental procedures were conducted in strict adherence to the animal experimentation guidelines of Sogang University and were approved by the institutional animal care and use committee (IACUCSGU 2022_08).

### Statistical analysis

The experimental results are displayed as the mean ± standard deviation (S.D.). The means of two experimental sets were compared using the unpaired Student’s *t*-test. Statistical evaluations were conducted using Microsoft Excel from Microsoft Corporation. A *p*-value less than 0.05 was considered to indicate statistical significance.

## Results

### KPT6566 treatment suppressed P19 cell growth, viability, colony-forming ability, and ATP production

**FIGURE 1:**
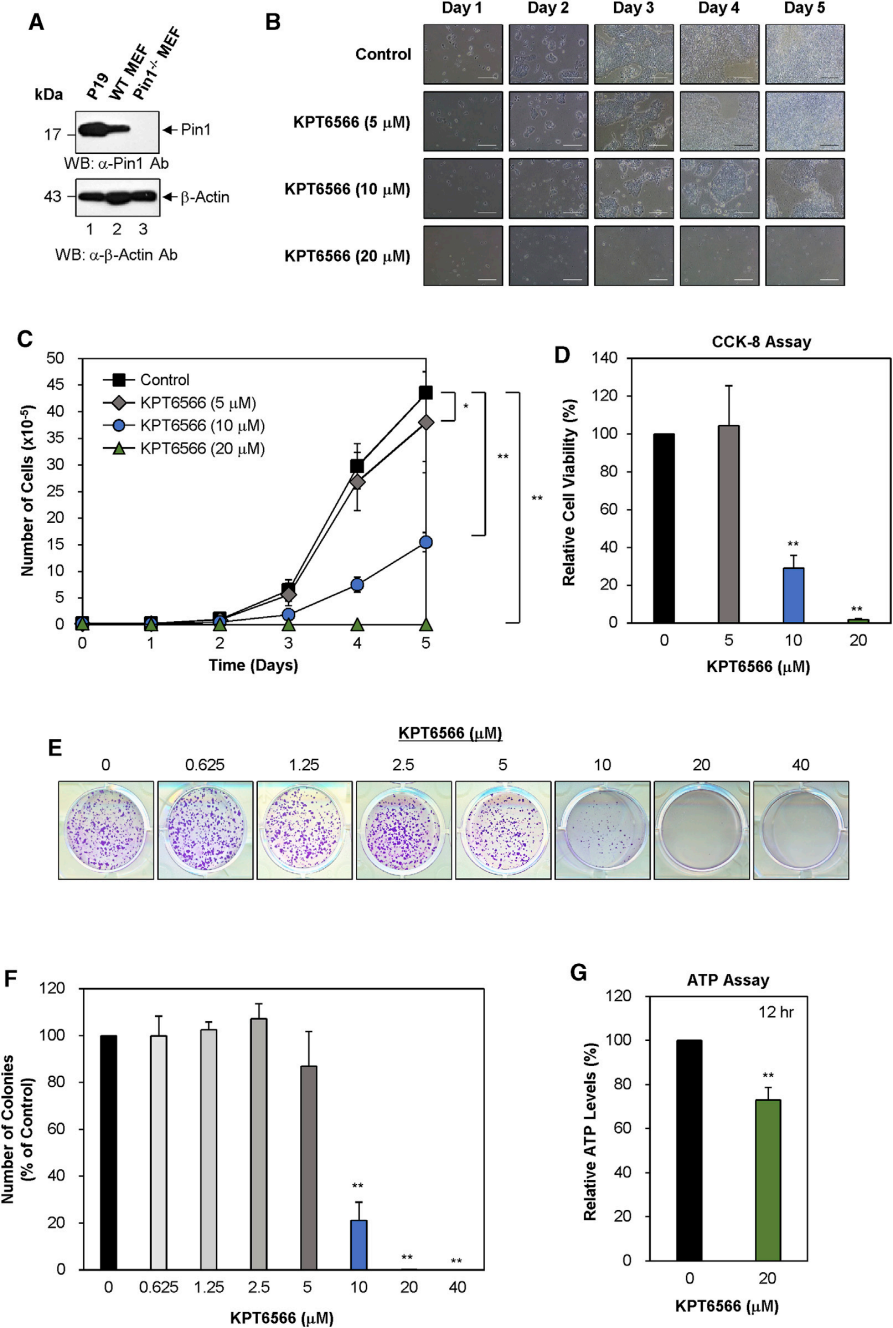
KPT6566 inhibition of P19 cell growth, viability, colony formation, and ATP production. **(A)** Analysis of Pin1 expression in mouse P19 embryonal carcinoma cells. To measure Pin1 expression in P19 cells, cultured cells were harvested for cell lysate preparation. Subsequently, total protein lysates were separated by SDS-PAGE using a 15% gel for Pin1 and a 12% gel for β-Actin, followed by transfer to a PVDF membrane for immunoblotting using either anti-Pin1 (upper panel) or β-Actin (lower panel) antibodies. Wild-type and Pin1^−/−^ MEFs were used as references for Pin1 protein in P19 cells. The molecular weight marker (New England Biolabs) size is denoted on the left in kilodaltons. **(B)** Morphological alterations in KPT6566-treated P19 cells. P19 cells were seeded in 12-well plates at a density of 2 × 10^4^ cells per well and cultured in DMEM with or without the predetermined KPT6566 concentration. Over a 5 day period, cellular morphology was assessed using an inverted phase-contrast microscope (IX71; Olympus) equipped with a 100 μm scale bar for precise measurements. **(C)** Effects of KPT6566 on P19 cell proliferation. P19 cells (2 × 10^4^) were exposed to KPT6566 at concentrations of 5, 10, and 20 μM. Cells were counted daily for 5 consecutive days using a hemacytometer. Data are expressed as mean ± standard deviation (S.D.). N = 6. *p < 0.05 and **p < 0.01 relative to control, unpaired Student’s t-test. **(D)** CCK-8 measurement of P19 cell viability after KPT6566 treatment. P19 cells were seeded at 2 × 10^3^ cells into 96-well plates and cultured in DMEM supplemented with vehicle (DMSO) or the indicated KPT6566 concentrations for 5 days prior to the assay. The CCK-8 assay was conducted on the fifth day post-KPT6566 treatment. Results are expressed as mean ± S.D. n = 9. **p < 0.01 relative to control. **(E)** Effect of KPT6566 on P19 colony formation. P19 cells were seeded at a density of 2.5 × 10^3^ cells per well and cultured in media containing vehicle control or a predetermined KPT6566 concentration. Following a 5 days treatment period with KPT6566, the capacity for colony formation was evaluated, and the resulting colonies were subsequently stained with 0.05% Crystal Violet to facilitate visualization. Representative images are shown. **(F)** Quantitative analysis of colony formation. Colonies were quantified using Image J. Data are expressed as percentage of control group (considered to be 100%). Data are expressed as mean ± S.D. n = 3. **p < 0.01 relative to control cells, unpaired Student's t-test. **(G)** Effect of KPT6566 treatment on ATP production in P19 cells. ATP production was measured in P19 cells 12 h post-treatment (DMSO or 20 μM KPT6566). KPT6566 inhibited ATP production by 27%. N = 5. **p < 0.01 relative to control cells, Student’s t-test.

Although Pin1 is overexpressed in multiple human cancers and cancer cell lines (Lu, 2003), its role in TGCTs remains poorly understood. Western blotting was used to measure Pin1 expression in embryonal carcinoma cells (P19 cell lysates). Wild-type and Pin1-null MEFs were used as controls for the presence of Pin1 protein. A Pin1 protein band was identified in both P19 cells (Figure 1A, top panel, labeled as P19) and wild-type MEFs (WT MEFs) and was absent in Pin1-null MEFs (Pin1^−/−^ MEFs). Notably Pin1 protein was more highly expressed in P19 cells than in wild-type MEFs. To normalize Pin1 protein levels, a β-actin was used as a loading control (Figure 1A, bottom panel).

To evaluate the potential effect of KPT6566 on P19 cell growth, 2 × 10^4^ P19 cells were seeded in 12-well plates and exposed to 5 μM, 10 μM, or 20 µM KPT6566. Over 5 days, cell morphology and growth were monitored using inverted phase-contrast microscopy. Untreated control P19 cells demonstrated robust growth and an epithelial cell-like morphology (Figure 1B top panels). Contrastingly, KPT6566 caused concentration- and time-dependent decreases in P19 cell proliferation (Figure 1B, second to bottom panels).

To investigate the effect of KPT6566 on cell proliferation, P19 cell counts were quantified at 24 h intervals over 5 days. KPT6566 treatment of P19 cells significantly attenuated cell proliferation in a concentration-dependent manner (Figure 1C).

A CCK-8 assay was used to assess cell viability. Consistent with total cell count findings (Figure 1C), KPT6566 significantly decreased the proliferative capacity of P19 cells (Figure 1D).

Colony formation ability is considered a surrogate indicator for the tumorigenic potential of cancer cells. To investigate the effect of KPT6566 on P19 cell clonal expansion, 2.5 × 10^3^ P19 cells were seeded into 12-well plates and exposed to a gradient of KPT6566 concentrations (0, 0.625, 1.25, 2.5, 5, 10, 20, and 40 μM) over a 5 day period. Subsequent Crystal Violet staining revealed that KPT6566 elicited a concentration-dependent decrease in the colony-forming ability of P19 cells (Figure 1E).

To quantify colony formation, P19 cell culture plate images were subjected to analysis using ImageJ software (https://imagej.nih.gov/ij/) (Figure 1F). Treatment with 0.625, 1.25, and 2.5 µM KPT6566 only modestly affected P19 cell colony formation capacity. Conversely, treatment with 5 μM and 10 µM KPT6566 significantly decreased colony numbers by 13% and 78%, respectively. Exposure to 20 μM and 40 µM KPT6566 nearly abrogated colony formation ability, with an approximate inhibition of 100%.

To investigate the role of KPT6566 in regulating P19 cell ATP production, intracellular ATP levels were measured in KPT6566-treated P19 cells. KPT6566 decreased ATP production by 27% reduction following 12 h of KPT6566 treatment (Figure 1G).

KPT6566 effectively triggers oxidative stress in different cell types (Campaner et al., 2017). Therefore, we examined if the level of reactive oxygen species (ROS) was increased in P19 cells treated with KPT6566. We employed the DCFDA (2′,7′–dichlorofluorescein diacetate) probe, which is sensitive to ROS, in a plate reader test to gauge the ROS level in P19 cells exposed to KPT6566. The ROS concentration was notably higher in P19 cells treated with KPT6566 than in untreated control cells (0 μM KPT6566) (Supplementary Figure S1A).

KPT6566 treatment significantly decreased cell proliferation, viability, colony-forming capacity, and ATP production in NCCIT cells.

**FIGURE 2:**
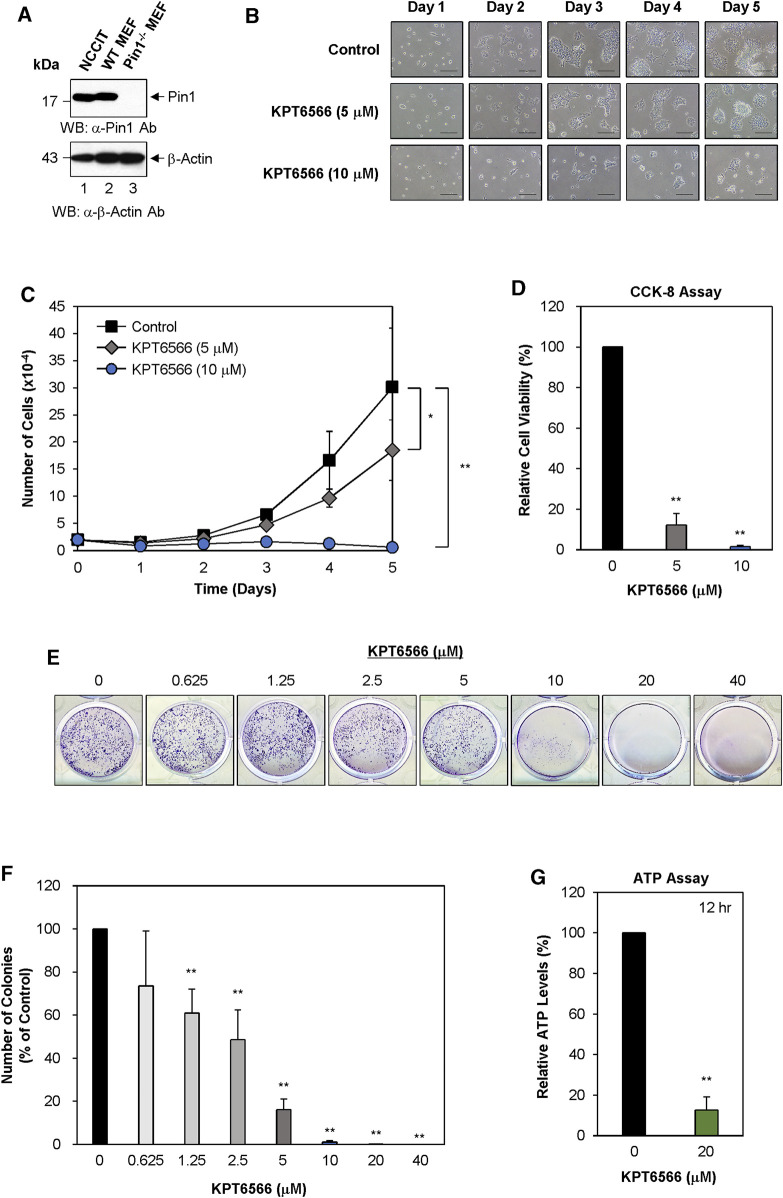
KPT6566 inhibition of NCCIT cell growth, viability, colony formation, and ATP production. **(A)** Measurement of Pin1 protein level in human NCCIT embryonal carcinoma cells. The investigation of Pin1 expression in NCCIT cells was conducted by harvesting cultured cells to obtain cell lysates. The total protein content was segregated utilizing SDS-PAGE, employing a 15% gel for anti-Pin1 and a 12% gel for anti-β-Actin immunoblots. Subsequent to transfer onto a PVDF membrane. Western blots were probed with anti-Pin1 (upper panel) or β-Actin (lower panel) antibodies. Wild-type and Pin1^−/−^ MEFs were used as controls for Pin1 expression. Presence in NCCIT cells. Notably, the molecular mass of human Pin1 (lane 1) was smaller than that of mouse Pin1 (lane 2). The molecular mass marker (New England Biolabs) size is delineated on the left in kilodaltons. **(B)** NCCIT cell morphology and proliferation following KPT6566 exposure. NCCIT cells cultivated in DMEM containing vehicle control or KPT6566 (5 or 10 μM). Cell growth was monitored over a 5 days period utilizing an inverted phase-contrast microscope (IX71; Olympus). Scale bar, 100 μm. **(C)** Effect of KPT6566 on NCCIT cell proliferation. NCCIT cells were exposed to KPT6566 (5 or 10 μM), followed by cell counting at 24 h intervals over 5 days. Data are presented as mean ± S.D. n = 4. *p < 0.05 and **p < 0.01 relative to DMSO control, unpaired Student’s t-test. **(D)** Effect of KPT6566 on cell viability in NCCIT cells. NCCIT cells were seeded at a density of 2 × 10^3^ cells per well in 96-well plates cultivated in DMEM with or without the specified concentrations of KPT6566 for 5 days. The CCK-8 assay was conducted on the fifth day post-KPT6566 treatment. KPT6566 significantly decreased NCCIT cell viability. Results are expressed as mean ± S.D. n = 7. **p < 0.01 relative to control. **(E)** Effect of KPT6566 on NCCIT colony formation. NCCIT cells were seeded at a density of 2.5 × 10^3^ cells per well and cultivated in media supplemented with vehicle or the specified KPT6566 concentrations for 5 days. After a 5 days incubation period, colony formation assays were conducted, and the resulting colonies were stained with 0.05% Crystal Violet for visualization. Representative images are shown. **(F)** Quantification of colony formation in KPT6566-treated NCCIT cells. Colonies were counted using ImageJ software, and the percentage of colonies relative to control (100%) was calculated. Data are presented as the mean ± S.D. n = 8. **p < 0.01 versus control cells, unpaired Student's t-test. **(G)** Effect of KPT6566 treatment on ATP production in NCCIT cells. ATP production was measured in NCCIT cells 12 h post-treatment (DMSO or 20 μM KPT6566). KPT6566 inhibited ATP by 87% relative to control (n = 6). **p < 0.01 versus control, unpaired Student’s t-test.

The potential inhibitory effects of KPT6566 on proliferation, viability, colony-forming ability, and ATP production in NCCIT cells were evaluated. NCCIT lysates were subjected to Western blot analysis to measure Pin1 expression. Wild-type and Pin1^−/−^ MEFs were utilized as controls, consistent with the methodology applied to P19 cells in Figure 1A. Pin1 protein was expressed in both NCCIT cells and wild-type MEFs (Figure 2A, top panel). Intriguingly, the molecular weight of human Pin1 in NCCIT cells was modestly decreased relative to mouse Pin1 in wild-type MEFs (Figure 2A, top panel). Human Pin1 consists of 163 amino acids, with a theoretical molecular weight of 18.24 kDa (https://www.uniprot.org/uniprot/Q13526#sequences), while mouse Pin1 is comprised of 165 amino acids with a theoretical molecular weight of 18.37 kDa (https://www.uniprot.org/uniprot/Q9QUR7#sequences). β-Actin was used as a loading control to normalize Pin1 protein levels (Figure 2A, bottom panel). We also compared Pin1 expression between NCCIT cells and multiple human cancer cell lines (Supplementary Figure S2).

To assess if Pin1 is involved in TGCTs, we examined the relationship between Pin1 expression and the survival rate of TGCT patients using data from The Cancer Genome Atlas (TCGA) database. Increased expression of Pin1 correlated with lower overall survival among 68 patients from TCGA. Kaplan–Meier survival analysis showed that patients with elevated Pin1 levels had a lower survival rate than those with lower Pin1 levels (Supplementary Figure S3A). To investigate the Pin1 expression profile in TGCT and standard tissue samples, we used gene expression profiling data from TCGA. Gene expression analysis of tumor and non-tumor samples from the TCGA cohort showed that *Pin1* mRNA expression was higher in TGCT samples than in non-tumor (normal testis) samples (Supplementary Figure S3B).

Subsequently, we examined the effect of KPT6566 on NCCIT cell proliferation. Twenty thousand NCCIT cells were seeded into 12-well plates and exposed to 5 or 10 µM KPT6566. Over a period of 5 days, cells were observed using inverted phase-contrast microscopy. Untreated NCCIT cells established continuous colonies and exhibited epithelial-like growth patterns (Figure 2B). Contrastingly, cell proliferation was decreased in KPT6566-treated NCCIT cells in a concentration- and time-dependent manner (Figure 2B).

To evaluate the effect of KPT6566 on the proliferative capacity of NCCIT cells, a time-course assessment was conducted, with cell counts measured at 24 h intervals over a duration of 5 days. Cell count was significantly decreased in KPT6566-treated cells (Figure 2C), suggesting that KPT6566 significantly inhibited NCCIT cell proliferation.

Cell viability was assessed using a CCK-8 assay. Consistent with decreased cell numbers (Figure 2C), CCK-8 cell viability analysis revealed that treatment with 5 or 10 μM KPT6566 significantly decreased the proliferative capacity of NCCIT cells (Figure 2D).

The effect of KPT6566 on clonal proliferation of NCCIT cells was examined. A total of 2.5 × 10^3^ NCCIT cells were seeded in 12-well plates and treated with varying concentrations of KPT6566 (0, 0.625, 1.25, 2.5, 5, 10, 20, and 40 µM). Following a 5 days incubation period, NCCIT cell colonies were stained with Crystal Violet for visualization. KPT6577 significantly decreased the colony-forming capacity of NCCIT cells (Figure 2E).

Colonies in each image were counted using ImageJ software. In NCCIT cells, treatment with KPT6566 at concentrations of 0.625, 1.25, 2.5, and 5 µM decreased colony numbers by 26%, 39%, 51%, and 84%, respectively (Figure 2F). Moreover, KPT6566 treatment at concentrations of 10 μM, 20 μM, and 40 µM substantially decreased colony formation ability, ranging from approximately 99%–100% inhibition (Figure 2F).

To investigate the potential effect of KPT6566 on ATP synthesis in NCCIT cells, intracellular ATP concentrations were quantified in KPT6566-treated NCCIT cells. Treatment with 20 μM KPT6566 (12 h) decreased ATP levels by 87% (Figure 2G).

To confirm the effects of KPT6566 on NCCIT cells, ROS generation was evaluated using a kit to detect ROS. Treatment with KPT6566 induced ROS generation in NCCIT cells (Supplementary Figure S1B).

### Concentration-dependent effect of KPT6566 on P19 and NCCIT cell proliferation *ex vivo*

**FIGURE 3:**
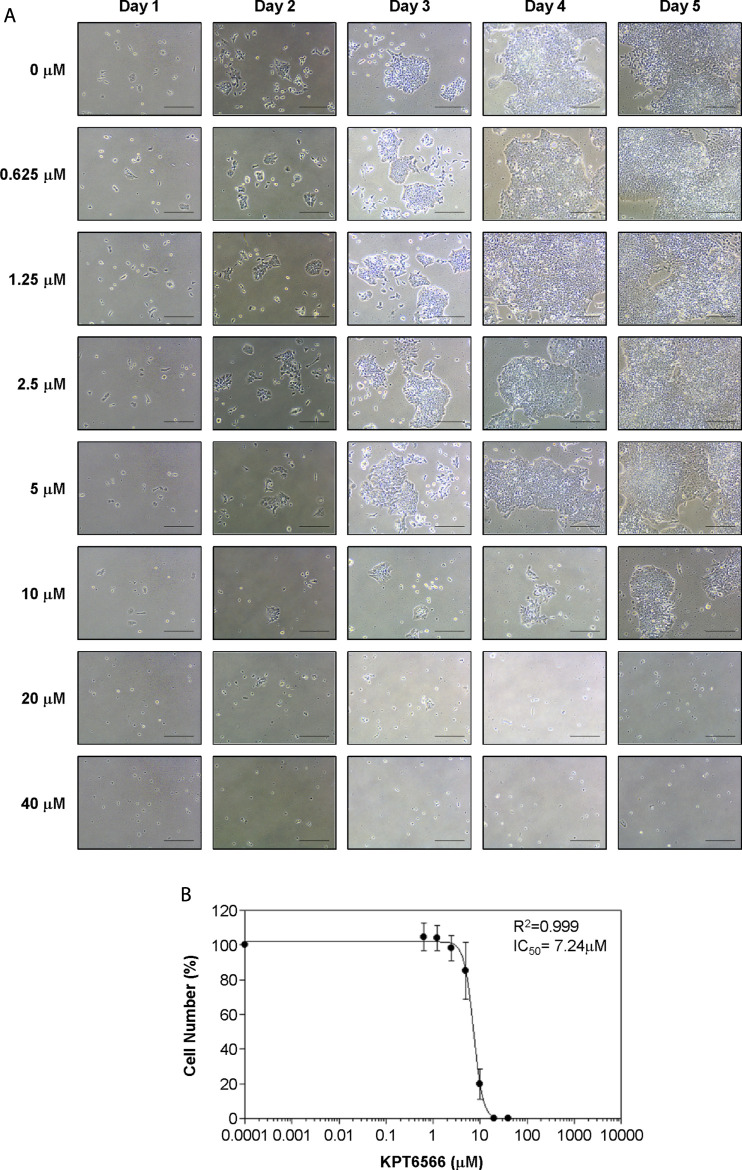
Concentration-dependent KPT6566 inhibition of P19 cell proliferation. **(A)** Morphological analysis of P19 cells treated with KPT6566. P19 cells were cultured in the presence or absence of the specified KPT6566 concentrations and monitored over a 5-day period using an inverted phase-contrast microscope. Scale bars, 100 µm. **(B)** Concentration-response relationship between KPT6566 concentrations and P19 cell proliferation. P19 cells were exposed to varying concentrations of KPT6566, and growth inhibition was assessed with cell counting. Cell counts are represented as percentage of control (DMSO, 0 μM KPT6566). Data are presented as mean ± S.D. derived from three independent experiments, each conducted in triplicate. The half-maximal inhibitory concentration (IC_50_) of KPT6566 was determined to be 7.24 μM in P19 cells (*R* ^2^ = 0.999, n = 3).

To determine the half-maximal inhibitory concentration (IC50) of KPT6566 in TGCTs, we examined its effect on P19 cell viability. P19 cell growth following KPT6566 treatment was assessed using microscopy (Figure 3A). KPT6566 decreased P19 cell proliferation, which was both concentration- and time-dependent. A sigmoidal concentration-response curve was generated using SOFTMAX PRO software to determine the IC_50_ of KPT6566, identifying an IC_50_ of 7.24 μM for P19 cells (Figure 3B).

**FIGURE 4:**
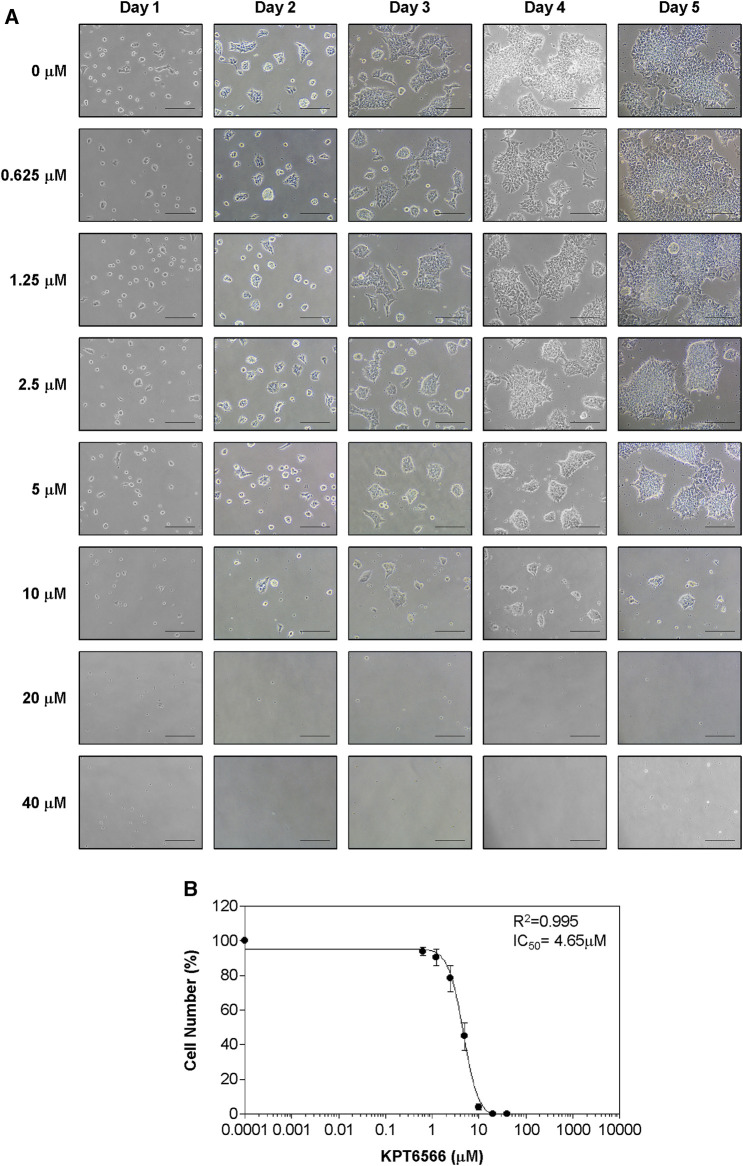
Concentration-dependent KPT6566 inhibition of NCCIT cell growth. **(A)** Morphological changes in NCCIT cells following KPT6566 exposure. Cells were treated with incremental concentrations of KPT6566 and examined using inverted phase-contrast microscopy over 5 days. Scale bars, 100 µm. **(B)** Suppression of NCCIT cell proliferation by KPT6566. NCCIT cells were exposed to incremental KPT6566 dosage, and growth inhibition was assessed with cell counting. Cell counts are represented as percentage of control (DMSO, 0 μM KPT6566). Data are expressed as mean ± S.D. from three independent experiments performed in triplicate. The IC_50_ value of KPT6566 in NCCIT cells was determined to be 4.65 µM (*R* ^2^ = 0.995, n = 3).

The effect of KPT6566 on NCCIT cell proliferation was examined. The progression of NCCIT cell proliferation post-KPT6566 treatment was observed with light microscopy (Figure 4A). KPT6566 treatment decreased NCCIT cell growth in a time- and concentration-dependent manner, with an IC_50_ value of 4.65 µM (Figure 4B). This value was lower than the IC_50_ of P19 cells (Figure 3B). These data suggested NCCIT cells were more sensitive to KPT6566 than were P19 cells.

### KPT6566 induced apoptotic cell death in P19 and NCCIT cells

**FIGURE 5:**
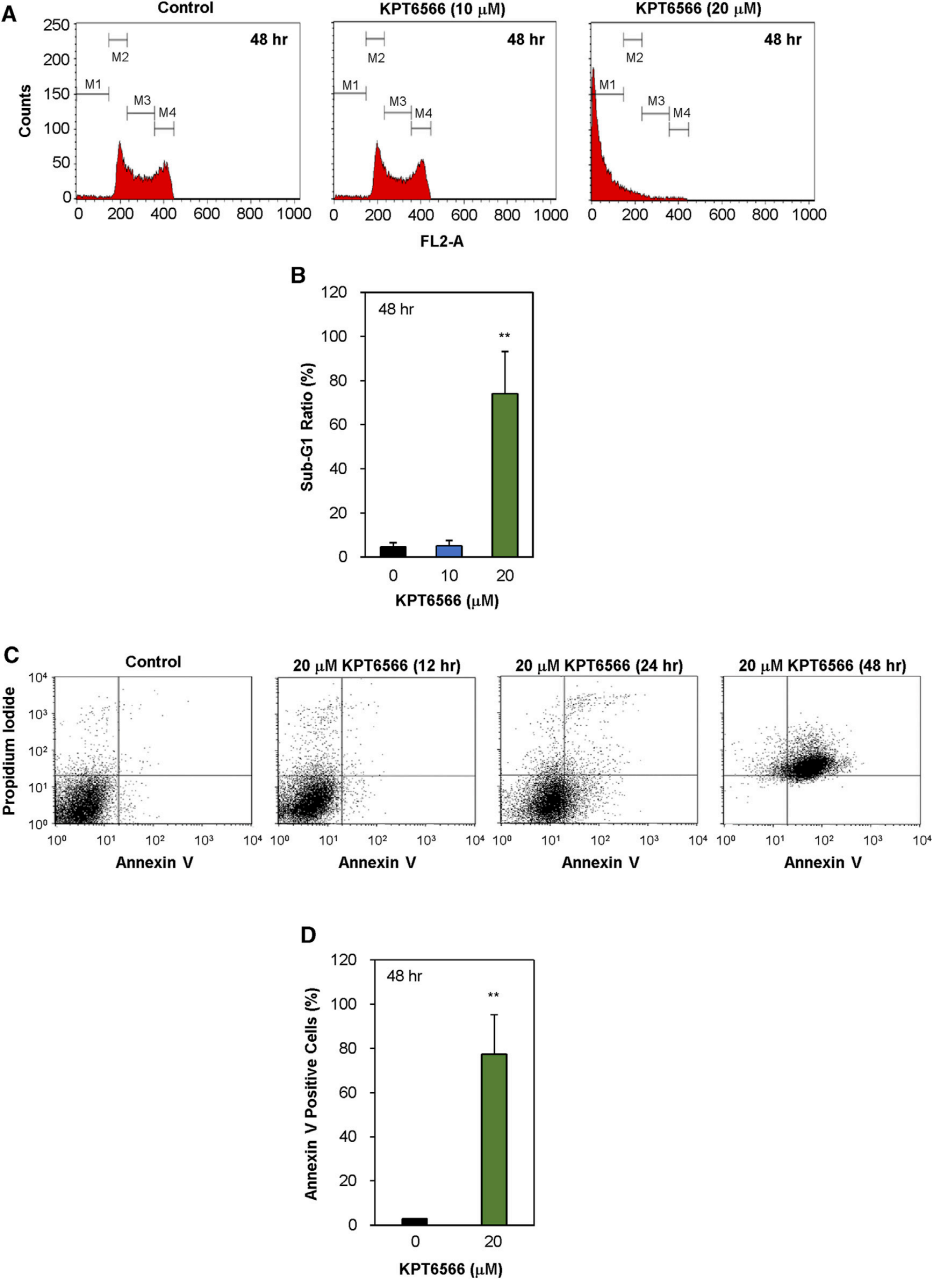
Apoptotic effects of KPT6566 in P19 cells. **(A)** Quantification of P19 cell cycle distribution post-KPT6566 treatment with flow cytometric analysis. P19 cells were cultured in the presence or absence of either 10 or 20 μM KPT6566 for 48 h and subjected to propidium iodide staining. The designated populations, denoted as M1, M2, M3, and M4, correspond to Sub-G1, G0/G1, S, and G2/M phases, respectively. Graphical representation of cell cycle distribution demonstrates induction of apoptosis in P19 cells subjected to KPT6566 treatment. **(B)** P19 cell accumulation in the sub-G1 phase following KPT6566 treatment. The percentage of P19 cells with sub-G1 DNA content was determined as a proportion of total cells analyzed. Data are expressed as mean ± S.D. **p < 0.01 versus control group (n = 6). **(C)** Annexin V-PI assessment of apoptosis. P19 cells were treated with 20 µM KPT6566 for 0, 12, 24, or 48 h. Subsequently, cells were stained with FITC-Annexin V-PI, and apoptosis was quantitatively analyzed using flow cytometry. **(D)** Percentage of apoptotic P19 cells following KPT6566 treatment. The percentage of apoptotic cells in P19 cell populations was assessed after KPT6566 treatment for 48 h. The apoptosis ratios for each experimental group are expressed as mean ± S.D. **p < 0.01 versus control group (n = 4).

Subsequently, the effect of KPT6566 on cell cycle distribution in P19 cells was investigated. P19 cells exposed to 10 µM or 20 µM KPT6566 for 48 h were stained with PI, and cell cycle distributions were analyzed using flow cytometry. Treatment with 20 µM KPT6566 significantly increased the proportion of P19 cells in the sub-G1 phase relative to the control group (Figure 5A), which was statistically significant, increasing from 4.6% in the control group to 74.2% in cells treated with 20 µM KPT6566 (Figure 5B). Conversely, treatment with 10 μM KPT6566 only marginally increased the sub-G1 phase population (5.2%) in comparison to control cells.

To investigate the potential association between decreased viability and apoptosis in cells treated with KPT6566, further Annexin V-FITC/PI staining experiments were conducted. Annexin V binds phosphatidylserine (PS), which is present in higher concentrations on the extracellular lipid bilayer of apoptotic cells due to membrane alterations that occur during apoptosis. Moreover, PI can be used to stain cells with compromised membrane integrity subsequent to apoptotic events (van Genderen et al., 2008; Lee et al., 2013). Following exposure to 20 μM KPT6566 for 12, 24, and 48 h, the proportions of apoptotic P19 cells were increased (Figure 5C). The proportion of apoptotic P19 cells (Annexin V-positive cells) was 1.8% after 12 h exposure to 20 μM KPT6566 (control Annexin V-positive cells = 1.4%). However, the apoptotic rate increased to 24.3% after 24 h exposure and reached 77.4% after 48 h, suggesting KPT6566 induced P19 cell apoptosis in a time-dependent manner. Quantifications of Annexin V-positive P19 cells after KPT6566 treatment for 48 h are shown in Figure 5D.

**FIGURE 6:**
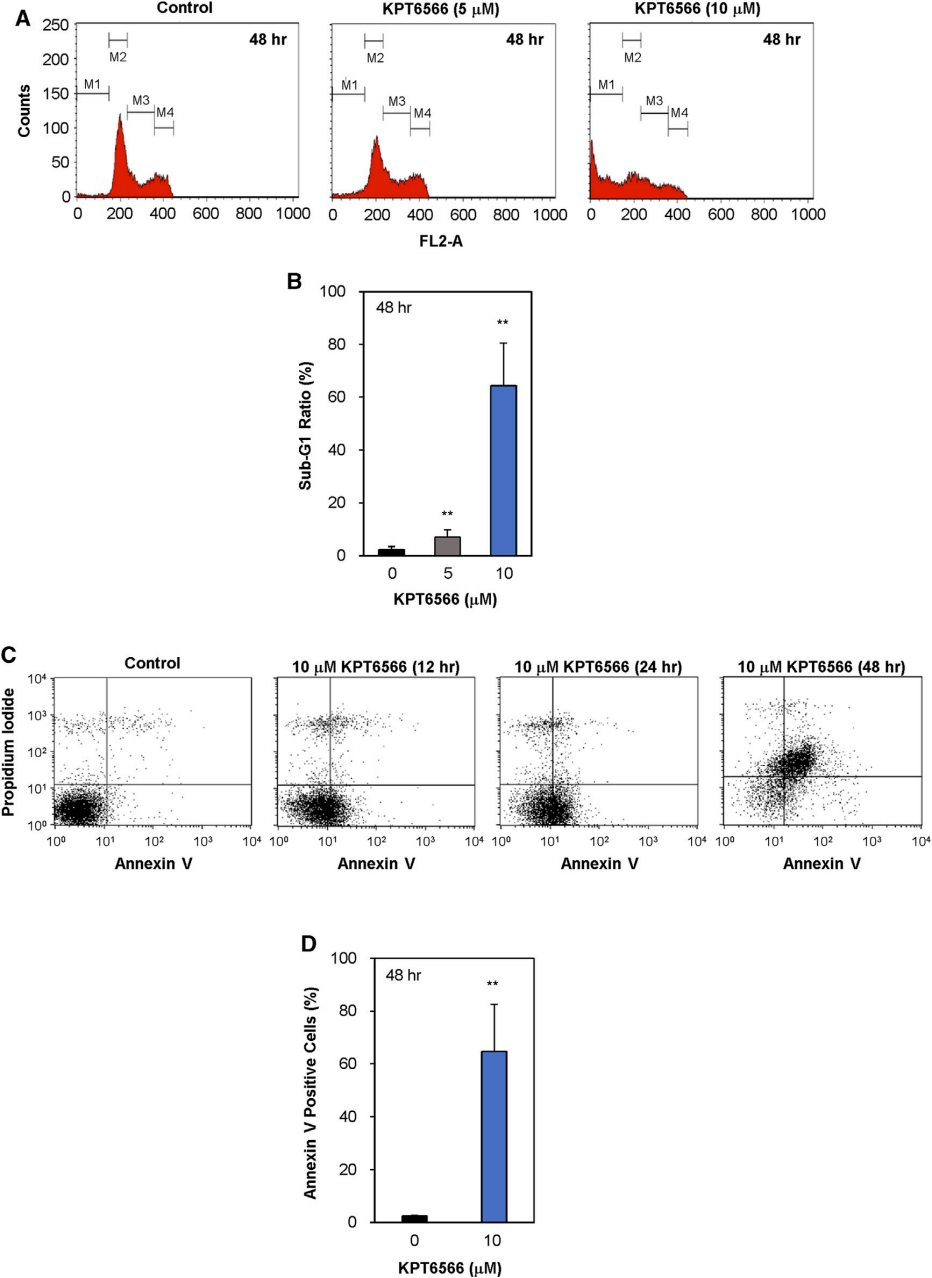
Apoptotic effects of KPT6566 in NCCIT cells. **(A)** Flow cytometric analysis of NCCIT cell cycle distribution following KPT6566 treatment. Cells were cultured with vehicle, 5 μM KPT6566, or 10 μM KPT6566 for 48 h and subjected to PI staining. The designations M1, M2, M3, and M4 correspond to the gated sub-G1, G0/G1, S, and G2/M populations, respectively. The histograms are representative images of cell cycle distribution, illustrating induction of apoptosis in NCCIT cells treated with KPT6566. **(B)** Accumulation of NCCIT cells in the sub-G1 phase following KPT6566 treatment. The percentage of NCCIT cells with sub-G1 DNA content was determined as a proportion of total cells analyzed. Data are expressed as mean ± S.D. **p < 0.01 versus control group. N = 4. **(C)** Annexin V-PI assessment of apoptosis. NCCIT cells were treated with 10 μM KPT6566 for 0, 12, 24, or 48 h, and subsequently stained with FITC-Annexin V-PI and analyzed with flow cytometry to measure apoptotic events. **(D)** Percentage of apoptotic NCCIT cells following KPT6566 treatment. Quantitative analysis of KPT6566-induced apoptosis in NCCIT cells demonstrates significantly increased apoptotic ratios. Data are expressed as mean ± S.D. n = 4. **p < 0.01 versus control group.

The effect of KPT6566 on cell cycle distribution of the NCCIT cell line was assessed. NCCIT cells were treated with either 5 or 10 µM of KPT6566 for 48 h and subsequently subjected to PI staining, followed by flow cytometric analysis of cell cycle distributions. KPT6566 increased the proportion of NCCIT cells in the sub-G1 phase in a concentration-dependent manner (Figure 6A). In Figure 6B, the quantification of the sub-G1 phase in NCCIT cells following 48 h KPT6566 treatment is illustrated. Treatment with both 5 and 10 µM KPT6566 significantly increased the proportion of cells in the sub-G1 phase, resulting in an increase from 2.3% (Control) to 7.0% (5 µM) and 64.4% (10 µM).

To investigate the potential association between decreased viability and apoptosis in cells treated with KPT6566, cells were double-stained with Annexin V-FITC/PI and subjected to flow cytometric analysis. Treatment of NCCIT cells with 10 µM KPT6566 for 12, 24, and 48 h significantly increased apoptosis in NCCIT cells (Figure 6C). The percentage of apoptotic (Annexin V-positive) NCCIT cells was increased ∼65% after 48-h exposure (Figure 6D). These findings suggest KPT6566 elicited apoptotic cell death in NCCIT cells in a time-dependent manner. Collectively, these findings demonstrated that KPT6566 decreased the numbers of viable cells and increased apoptotic cell populations in both P19 and NCCIT embryonal carcinoma cell lines.

### KPT6566 decreased expression of Oct-4 and Sox2 in P19 and NCCIT cells

**FIGURE 7:**
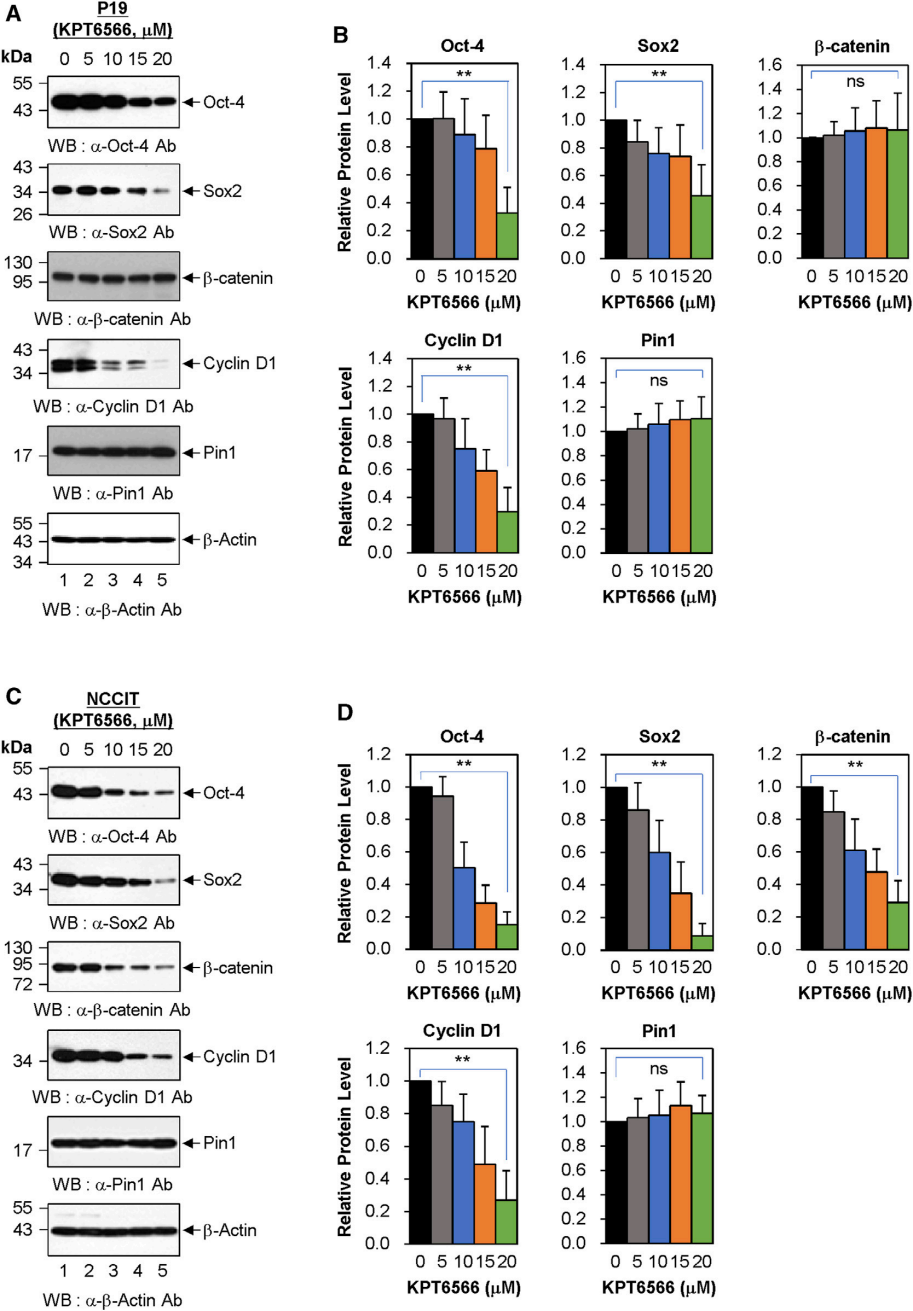
Effect of KPT6566 on embryonic transcription factor levels in P19 and NCCIT Cells. **(A)** Western blot measurement of Pin1 target proteins in P19 cells. P19 cells were treated with the indicated concentrations of KPT6566 for 48 h. The lysates were then separated by SDS-PAGE, utilizing 12% gels for Oct-4, β-catenin, and Cyclin D1; 15% gels for Sox2 and Pin; and 12% gel for β-Actin. Protein levels were measured by probing with antibodies against Oct-4, Sox2, β-catenin, Cyclin D1, Pin1, and β-Actin. β-Actin was used as a loading control. **(B)** Quantitative analysis of protein levels in P19 cells following KPT6566 treatment. Bar graphs display mean ± S.D. of relative intensities for Oct-4 (n = 8), Sox2 (n = 5), β-catenin (n = 8), Cyclin D1 (n = 5), and Pin1 (n = 8) bands, normalized to β-Actin. Nonsignificant (ns). **p < 0.01 relative to control, unpaired Student's t-test. **(C)** Western blot measurement of Pin1 target proteins in NCCIT cells. NCCIT cells were treated with the specified KPT6566 concentrations for 48 h. The extracts were resolved by SDS-PAGE (12% gel for Oct-4, 15% gel for Sox2, 12% gel for β-catenin, 12% gel for Cyclin D1, 15% gel for Pin1, and 12% gel for β-Actin) and transferred to a PVDF membrane. Protein levels were measured by immunoblotting for anti-Oct-4, anti-Sox2, anti-β-catenin, anti-Cyclin D1, anti-Pin1, and anti-β-Actin antibodies. β-Actin was used as a loading control. **(D)** Quantitative analysis of protein levels in NCCIT cells following KPT6566 treatment. Protein levels of Oct-4, Sox2, β-catenin, Cyclin D1, and Pin1 proteins in NCCIT cells were quantified after treatment with the specified concentrations of KPT6566. Bar charts depict mean ± S.D. of the relative intensities of Oct-4 (n = 6), Sox2 (n = 6), β-catenin (n = 6), Cyclin D1 (n = 5), and Pin1 (n = 6) bands normalized to β-Actin. Ns = nonsignificant, *p* > 0.05. **p < 0.01 relative to control, unpaired Student’s t-test.

Subsequently, we examined the mechanism by which KPT6566 suppressed cell growth and induced apoptosis in P19 and NCCIT cells. P19 cells were exposed to 5, 10, 15, and 20 µM KPT6566 for 48 h, and potential targets for KPT6566 were subsequently investigated. Interestingly, KPT6566 treatment downregulated embryonic transcription factors such as Oct-4 and Sox2 in P19 cells (Figure 7A). Additionally, KPT6566 treatment decreased Cyclin D1 levels in P19 cells (Figure 7A). However, KPT6566 treatment did not affect β-catenin protein levels in P19 cells (Figure 7A). Moreover, although a prior study reported that KPT6566 degrades Pin1 protein following covalent binding with its catalytic domain (Campaner et al., 2017), in the present study we identified that Pin1 levels were not affected by KPT6566 treatment (Figure 7A). Western blots were normalized using β-Actin as a loading control (Figure 7A). Quantitative analyses demonstrated that KPT6566 treatment significantly decreased protein levels of embryonal transcription factors Oct-4 and Sox2 and cell cycle regulator Cyclin D1 in P19 cells (Figure 7B). However, protein levels of β-catenin and Pin1 were not affected by any of the KPT6566 concentrations tested (Figure 7B).

The effects of KPT6566 on protein levels in NCCIT cells were also investigated. Notably, KPT6566 decreased protein levels of Oct-4, Sox2, and Cyclin D1 proteins in NCCIT cells (Figure 7C). Intriguingly, in contrast to its effects on P19 cells, KPT6566 treatment decreased β-catenin protein levels in NCCIT cells (Figure 7C). However, consistent with findings in P19 cells, KPT6566 treatment did not affect Pin1 protein levels in NCCIT cells (Figure 7C). Quantifications of Western blot analyses following treatment with KPT6566 in NCCIT cells are shown in Figure 7D.

Although it has been reported that KPT6566 treatment induces Pin1 degradation in PC-3, PANC-1, and H1299 cells (Campaner et al., 2017), our results showed no evidence of Pin1 degradation after KPT6566 treatment in P19 and NCCIT cells. Similarly, we previously showed that treatment with KPT6566 does not affect the level of Pin1 protein in Caco-2 colon cancer cells (Kim et al., 2022). To confirm that KPT6566 does not induce Pin1 degradation in P19 cells, a series of cycloheximide (CHX) chase assays were performed in these cells. Even when protein synthesis was inhibited by CHX, Pin1 expression was not significantly diminished in P19 cells treated with KPT6566 for 36 h (Supplementary Figures S4A, B). Cyclin D1 was used as a positive control to confirm that CHX and KPT6566 worked properly. Similar results were obtained in experiments using NCCIT cells (Supplementary Figures S4C, D), suggesting that KPT6566 reduces the Pin1 level in a tumor cell type- and context-dependent manner.

The effect of KPT6566 on the Pin1 level was also investigated after treatment with MG-132 in P19 cells. After KPT6566 treatment, the Cyclin D1 level was rescued by MG-132, but the Pin1 level was slightly increased or almost the same upon MG-132 treatment in P19 cells (Supplementary Figure S4E). The results obtained using NCCIT cells were similar to those obtained using P19 cells (Supplementary Figure S4F).

We next examined whether KPT6566 controls the peptidyl-prolyl isomerase (PPIase) activity of Pin1 in TGCT cells (Supplementary Figure S5). To measure the endogenous PPIase activity of Pin1, P19 cells were treated with 20 μM KPT6566 for 48 h and Pin1 activity was monitored using a SensoLyte^®^ Green Pin1 Activity Assay Kit. KPT6566 treatment markedly downregulated Pin1 activity in P19 cells (more than 75% inhibition) (Supplementary Figure S5B). Experiments performed using NCCIT cells also showed that treatment with 20 μM KPT6566 for 48 h inhibited Pin1 activity by about 70% (Supplementary Figure S5E). These data indicate that treatment of P19 and NCCIT cells with KPT6566 inhibited endogenous Pin1 activity, without stimulating degradation of Pin1 protein.

We subsequently examined the gene expression patterns of Oct-4 and Sox2 in P19 and NCCIT cells following suppression of Pin1 by treatment with 20 µM KPT6566 for 48 h. RT-PCR analysis showed that Pin1 suppression did not notably decrease the mRNA levels of *Oct-4* and *Sox2* in P19 cells (Supplementary Figure S6A–S6C). Similar results were obtained using NCCIT cells (Supplementary Figures S6D, F). Collectively, these findings indicate that while KPT6566 influences the protein stability of Oct-4 and Sox2, it does not affect their transcription in P19 and NCCIT cells.

### KPT6566 decreased P19 xenograft size in nude mice

**FIGURE 8:**
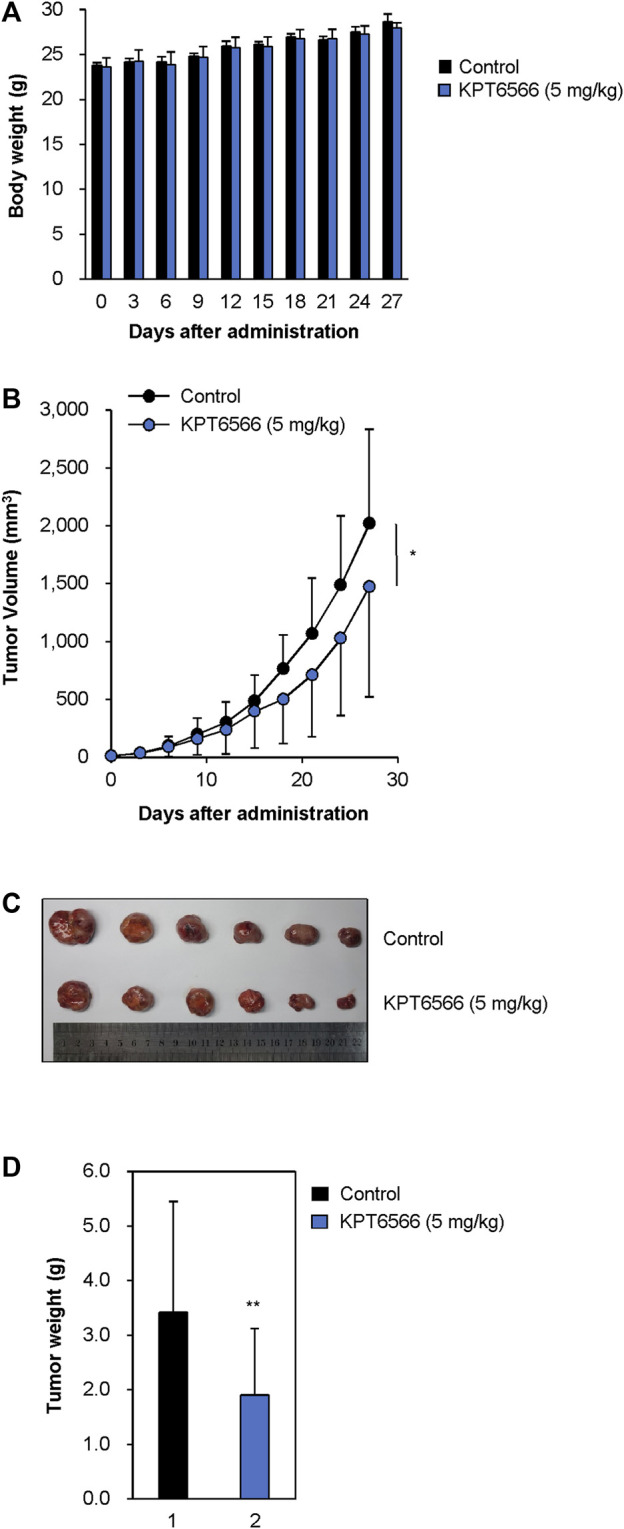
KPT6566 suppression of P19 cell tumor formation. **(A)** Average body weights of athymic mice following KPT6566 administration. During the study, mouse body weights were monitored to evaluate potential systemic toxicity of KPT6566. No significant reduction in body weight was observed. N = 6. **(B)** Decreased P19 cell tumor volume following KPT6566 treatment. Mice bearing P19 cell tumors were injected intraperitoneally with either vehicle or KPT6566 (5 mg/kg) at 3 days intervals over a 27 days period. Tumor dimensions were monitored throughout the experiment to determine the effects of KPT6566 treatment. Data are expressed as mean ± S.D. n = 6. *p < 0.05 versus control group, unpaired Student’s t-test. **(C)** Excised tumor sizes. P19 cell tumors were excised from mice treated with either vehicle control or KPT6566. Images were obtained 27 days post-tumor formation for comparative evaluation. **(D)** Evaluation P19 cell tumor weight following KPT6566 treatment. Weights of P19 tumors from either control or KPT6566-treated groups were quantified and presented as mean ± S.D. n = 6. **p < 0.01 versus control group, unpaired Student’s t-test.

The effect of KPT6566 on P19 cell tumorigenesis was investigated in a xenograft mouse model. Approximately 1 × 10^7^ P19 cells were suspended in 100 μL PBS and injected into the flanks of 8-week-old nude mice. When tumors reached 15–25 mm^3^, tumor-bearing nude mice were randomly divided into two groups, which were intraperitoneally injected every 3 days with either KPT6566 (5 mg/kg) or vehicle control for a total duration of 27 days. The health status of the KPT6566-treated mice was monitored by recording body weight every 3 days throughout the study. Mice treated with KPT6566 had comparable body weights to control animals (Figure 8A). No significant body weight loss occurred in the KPT6566-treated groups, indicating that KPT6566 did not have significant systemic toxicity in this experimental context.

Subsequently, we investigated the effect of KPT6566 on neoplastic proliferation *in vivo* by measuring tumor volumes through the course of the study. Consistent with *in vitro* findings, KPT6566 treatment significantly decreased tumor volume relative to the control group (Figure 8B). These findings suggested that Pin1 is integral to the tumorigenic potential of P19 cells, and that suppression of Pin1 activity markedly impeded tumor growth *in vivo*. At the experimental endpoint, intact tumor volume was decreased 27.0% in the KPT6566-treated group (1,476.3 ± 952.5 mm^3^) relative to vehicle control (2023.4 ± 809.8 mm^3^). In excised tumors, KPT6566 significantly decreased tumor size (Figure 8C). Mean tumor weight was decreased in the KPT6566-treated group (1.91 ± 1.21 g) relative to the control group (3.42 ± 2.03 g) (Figure 8D). Tumor weight was decreased by 44.2% in the KPT6566-treated group relative to the control group at the experimental endpoint (Figure 8D). These findings suggest a pivotal role for Pin1 in P19 cell tumorigenesis as Pin1 inhibition with KPT6566 effectively inhibited *in vivo* growth of P19 tumors.

## Discussion

**FIGURE 9:**
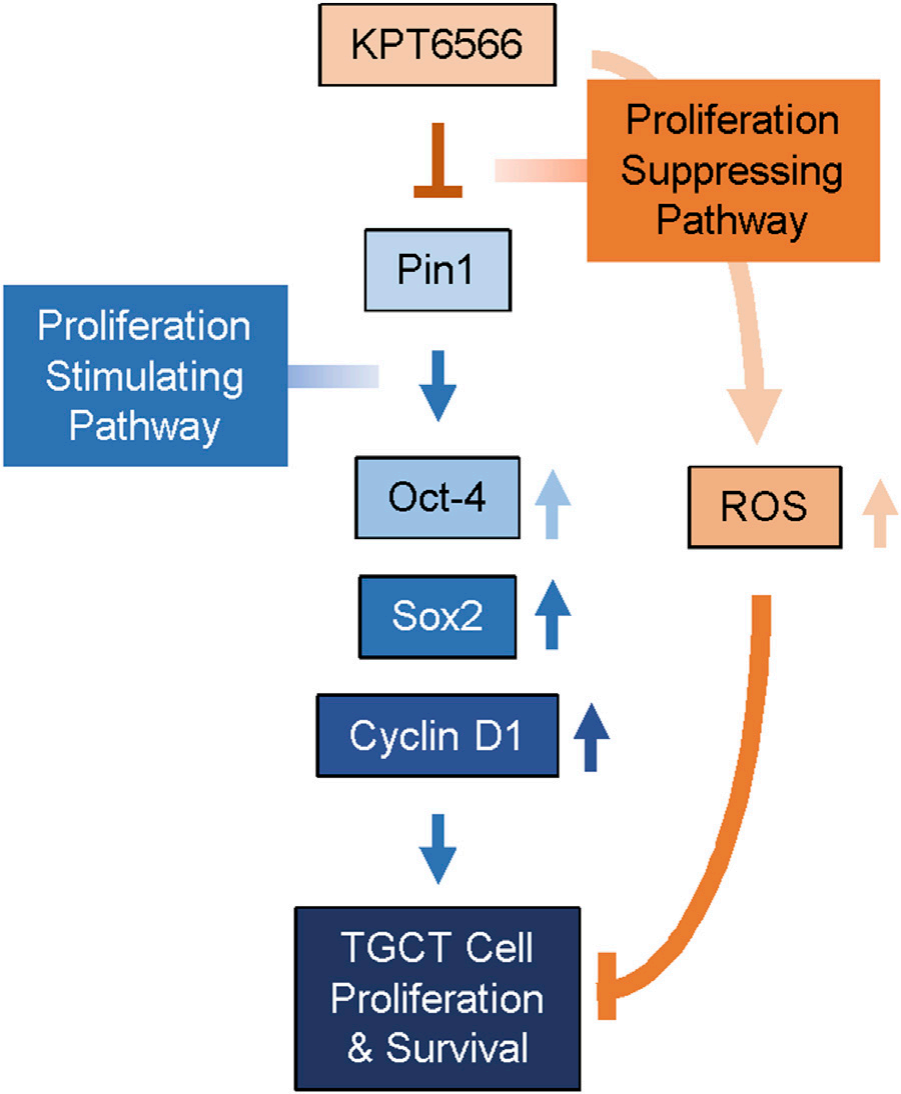
Proposed mechanism for KPT6566 suppression of TGCT cell proliferation. Pin1 augments expression of Oct-4, Sox-2, and Cyclin D1, facilitating proliferation of TGCT cells. KPT6566 inhibition of Pin1 decreases expression of Oct-4, Sox2, and Cyclin D1, increases cellular ROS generation, and impedes cell cycle progression, culminating in apoptotic cell death.

Multiple studies have identified that Pin1 contributes to the progression of diverse human cancers, but its role in TGCT had not previously been determined. TGCTs are the most common tumors in adolescent and young adult males. Increasing prevalence of TGCTs has prompted investigation of the underlying biological and genetic mechanisms of disease. In the present study, we determined whether the small-molecule Pin1 inhibitor KPT6566 affected viability and tumorigenic potential of embryonal carcinoma cells. We demonstrated that Pin1 inhibition controlled development of TGCTs. Both P19 and NCCIT cells expressed Pin1. In both cell lines, KPT6566 suppressed cell proliferation and colony-forming ability; induced apoptotic cell death; increased cellular ROS levels; and decreased Pin1 target protein levels, including the embryonal transcription factors Oct-4 and Sox2. Additionally, KPT6566 significantly decreased the tumorigenic potential of P19 cells *in vivo* in a nude mouse xenograft model. These findings suggest that targeting Pin1 is potentially an effective therapeutic approach for treatment of TGCTs. Further, the findings suggest that KPT6566 inhibition of Pin1 suppresses TGCT tumorigenesis by downregulating Oct-4 and Sox2 (Figure 9).

Although cisplatin is effective in most TGCTs, treatment of cisplatin-resistant TGCTs is a significant unmet clinical need. A substantive proportion of patients are cisplatin nonresponders, and in some cases chemoresistance develops, which in both contexts frequently results in mortality (Einhorn, 2002; Masters and Koberle, 2003; Adra and Einhorn, 2017). Because prior efforts to integrate more advanced targeted therapies for refractory TGCTs have been unsuccessful, identification of novel therapeutic modalities that are effective in cisplatin nonresponders is an urgent priority. Our current data demonstrate potent anticancer activity of the Pin1 inhibitor KPT6566 in TGCT. KPT6566 induced apoptotic cell death (Figures 5, 6) and inhibited tumorigenicity of TGCT cells (Figure 8), suggesting that targeting Pin1 in TGCTs is a promising potential approach to TGCT treatment. Pin1 is often overexpressed and activated in human cancers and regulates multiple oncogenic signaling pathways, so is a promising potential therapeutic target for TGCTs. This could lead to development of novel therapeutic strategies to address presently unmet clinical needs for TGCT treatment.

KPT6566 selectively inhibits Pin1, which influences the turnover and activity of oncogenic and tumor-suppressor proteins in human cancer cells. To evaluate the effect of KPT6566 on Pin1 targets, P19 and NCCIT cells were treated with KPT6566, followed by measurement of target protein levels by immunoblotting. Notably, KPT6566 significantly decreased Oct-4 and Sox2 protein levels in P19 and NCCIT cells (Figure 7). Oct-4 and Sox2 are essential embryonic transcription factors primarily involved in pluripotent cell regulation during early embryonic development and in embryonic and induced pluripotent stem cells (Nichols et al., 1998; Kamachi et al., 2000; Avilion et al., 2003; Boyer et al., 2005; Episkopou, 2005; Takahashi and Yamanaka, 2006; Takahashi et al., 2007). Further, Oct-4 and Sox2 activate cancer stem cells or tumor-initiating cells in some contexts (Leis et al., 2012; Wuebben and Rizzino, 2017; Zhang et al., 2020; Robinson et al., 2021). Although follow-up studies are necessary to determine the potential synergistic effects of Oct-4 or Sox2 inhibition with Pin1 inhibition, simultaneous targeting of these proteins could augment the efficacy of KPT6566 or other Pin1 inhibitors. These findings could provide valuable experimental support for development of KPT6566-based TGCT therapy.

Figures 1G, 2G demonstrated that ATP production was reduced in P19 and NCCIT cells treated with KPT6566. KPT6566 likely directly or indirectly influences the mitochondrial electron transport chain, resulting in diminished oxidative phosphorylation and, consequently, decreased ATP synthesis. Another plausible scenario is that KPT6566 disrupts glycolytic enzymes, leading to a decline in glycolysis and, consequently, a reduction in ATP generation. Furthermore, it is conceivable that KPT6566 elevates ATP-demanding processes, consequently diminishing overall ATP levels. For instance, KPT6566 treatment might activate cellular stress responses or repair mechanisms that necessitate additional ATP. Further investigations are imperative to elucidate the precise mechanism responsible for the decrease in ATP production following KPT6566 treatment in TGCTs.

Cyclin D1 and β-catenin are well-established Pin1 target proteins (Lu and Zhou, 2007; Liou et al., 2011; Kim et al., 2022). Consistent with prior studies, KPT6566 treatment decreased Cyclin D1 levels in both P19 and NCCIT cells (Figure 7). Intriguingly, the effects of KPT6566 on β-catenin protein degradation were cell line-dependent: KPT6566 decreased β-catenin in NCCIT cells but not P19 cells. Further, although prior findings demonstrated that KPT6566 selectively targets Pin1 for degradation following covalent binding (Campaner et al., 2017), KPT6566 did not affect Pin1 levels in either P19 or NCCIT cells. Thus, these findings imply that inhibition of Pin1 activity via covalent binding to its catalytic domain and proteasomal Pin1 degradation could be distinct, separable processes, at least in the context of the cell lines tested in the present study. Additional studies are needed to further elucidate these processes.

Numerous Pin1 inhibitors have been reported thus far, which have targeted both covalent and noncovalent inhibition strategies (Moore and Potter, 2013; Zhou and Lu, 2016; Yu et al., 2020). However, despite significant efforts, the efficacies of previously identified Pin1 inhibitors have been limited due to insufficient specificity, potency, and *in vivo* stability. The small-molecule KPT6566 has unique chemical properties and specificity, suggesting this inhibitor is a promising candidate for potential use as an anticancer drug (Campaner et al., 2017). The unique chemical properties of KPT6566 enable targeting of specific cancer cells, increasing its efficacy while decreasing possible side effects. However, the efficacy of KPT6566 could potentially be improved by further structural optimization and modifications. Refining the molecular structure of KPT6566 could improve its druglikeness, which refers to characteristics that enable use of a small molecule as an anticancer drug. Improving druglikeness includes optimization of important parameters such as solubility, stability, and bioavailability, which influence how effectively the molecule is absorbed, distributed, metabolized, and excreted by the body. These strategies could enable successful development of KPT6566 or related compounds as safe and effective anticancer drugs for human use.

In summary, our findings suggest that inhibiting Pin1 activity could be a promising therapeutic approach for TGCTs. Pin1 contributes to TGCT cell proliferation and survival by increasing protein levels of the pluripotency-associated transcription factors Oct-4 and Sox-2 and cell cycle regulator Cyclin D1. These properties are crucial for maintaining the pluripotency, self-renewal capacity, and cell cycle progression of TGCT cells. Considering the role of Pin1 in TGCT cell proliferation and its contribution to the aggressiveness of TGCTs, targeting Pin1 could disrupt molecular pathways that drive tumor growth and progression. Pin1 inhibition would decrease protein levels of Oct-4, Sox-2, and Cyclin D1, suppressing TGCT cell self-renewal and inhibiting cell cycle progression. Ultimately, these processes induce apoptosis in TGCT cells. Therefore, development of therapeutic strategies that specifically target Pin1 could provide a novel and effective treatment option for patients with TGCTs, particularly those with aggressive, nonseminomatous germ cell tumors. Further research and clinical trials would be necessary to evaluate the efficacy and safety of Pin1-targeting therapies and to determine their potential role in clinical management of TGCTs.

## Data Availability

The original contributions presented in the study are included in the article/Supplementary Material, further inquiries can be directed to the corresponding author.
