# Supplementary material for: KPT6566 induces apoptotic cell death and suppresses the tumorigenicity of testicular germ cell tumors
Source: Front Cell Dev Biol. 2023 Nov 2;11:1220179. doi: 10.3389/fcell.2023.1220179 (PMC10652286; doi:10.3389/fcell.2023.1220179)
Supplement: Supplementary file 1 [file DataSheet1.PDF]

KPT6566 Induces Apoptotic Cell Death and Suppresses the Tumorigenicity of Testicular Germ Cell Tumors

Ruijing Sun, Eun Joo Lee, Seonock Lee, Gamin Kim, and Jungho Kim

Supplementary Material

Supplementary Materials and Methods

Cell culture

In specific experiments, the culture medium was supplemented with 10 μg/mL cycloheximide (Sigma-Aldrich), 10 μM MG-132 (Sigma-Aldrich), or 5 or 20 μM KPT6566.

Reactive oxygen species (ROS) assay

Cellular ROS levels were measured using a DCFDA Cellular ROS Detection Assay Kit (Abcam) according to the manufacturer's instructions. Fluorescence emitted by DCF (2',7'-dichlorofluorescein) was captured using an Enspire 2300 Multilabel Plate Reader (Perkin Elmer) set at excitation and emission peaks of 485 and 535 nm, respectively.

Pin1 activity assay

Pin1 activity was assessed using a SensoLyte® Green Pin1 Kit as per the instructions provided by AnaSpec, Inc. Briefly, lysates of P19 or NCCIT cells (20 μL, 1.5 μg/μL) were mixed with the Pin1 substrate (50 μL) and the Pin1 developer solution (30 μL) and placed into a 96-well plate. Pin1 activity was then evaluated by noting the fluorescence levels at excitation/emission = 490/520 nm with an Enspire 2300 Multilabel Plate Reader.

Reverse transcription (RT)-PCR

Total RNA was isolated from P19 and NCCIT cells using TRIzol solution (Invitrogen). Subsequently, cDNA was synthesized using the Superscript First-strand Synthesis System (Invitrogen) and then RT-PCR was performed to amplify *Oct-4* and *Sox2*. β-*Actin* mRNA was utilized as an internal control. The following primers were used: mouse *Oct-4*, 5′-ATGGCTGGACACCTGGCTTCAGAC-3′ and 5′-ACAGTATGCCATCCCTCCGCAGAAC-

3′; mouse *Sox2*, 5’-TTAACGCAAAAACCGTGATG-3’ and 5’-GAAGCGCCTAACGTACCACT-3’; human *Oct-4*, 5’-CTCCTGGAGGGCCAGGAATC-3’ and 5’-CCACATCGGCCTGTGTATAT-3’; human *Sox2*, 5’-GCACATGAACGGCTGGAGCAACG-3’ and 5’-TGCTGCGAGTAGGACATGCTGTAGG-3’; and human and mouse β-*actin*, 5′-GCTCGTCGTCGACAACGGCTC-3′ and 5′-CAAACATGATCTGGGTCATCTTCTC-3′.

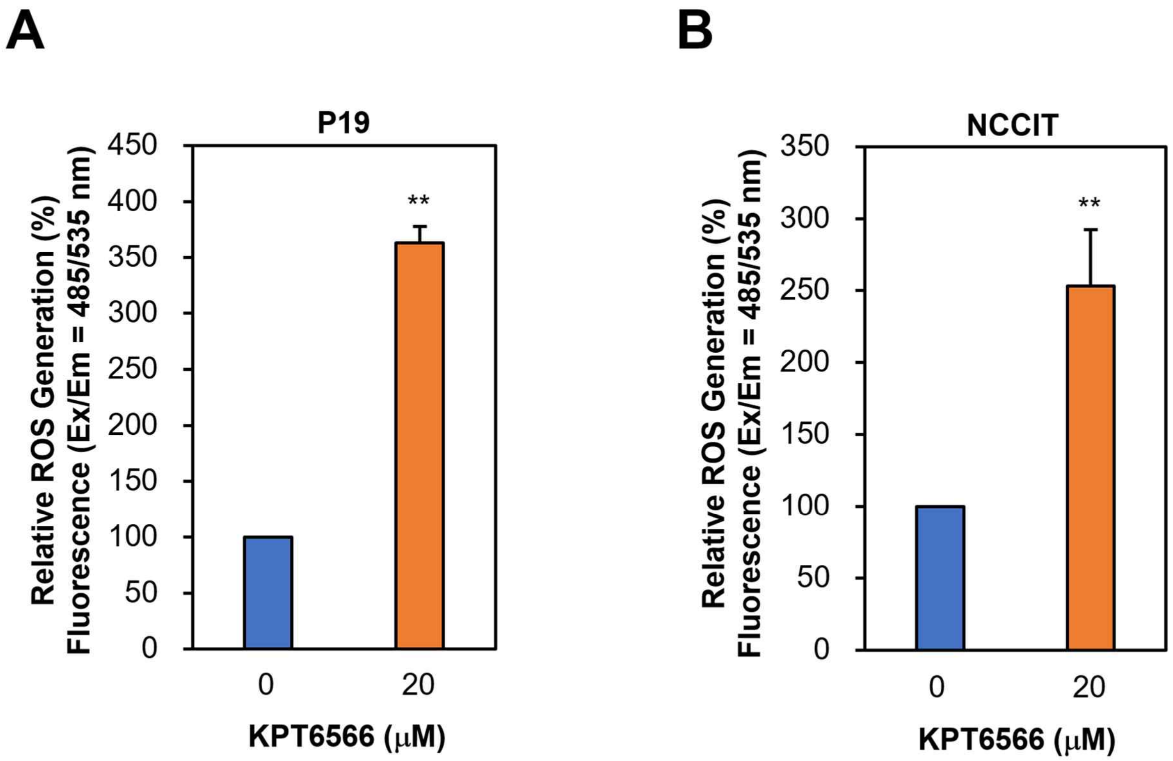

Supplementary Figure S1. Induction of ROS production by KPT6566 in P19 (A) and NCCIT (B) cells. After treating P19 or NCCIT cells with 20 μM KPT6566 for 48 hours, cellular ROS levels were assessed using a ROS detection assay kit. Values represent mean ± S.D. (n = 5). ***p* < 0.01.

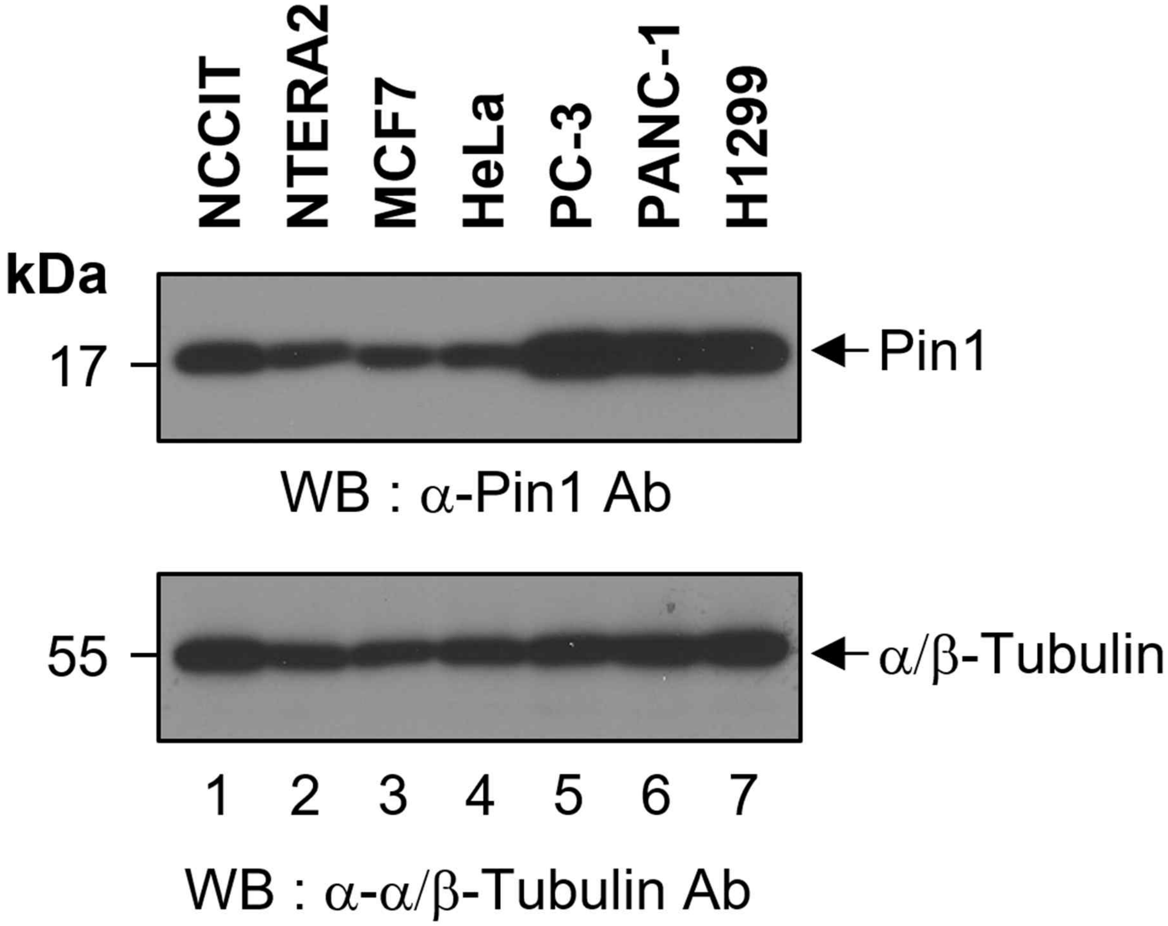

Supplementary Figure S2. Comparative analysis of Pin1 expression in NCCIT cells and multiple human cancer cells. Western blot analysis was performed to compare Pin1 expression between the NCCIT cell line (lane 1) and other cancer cell lines. The cancer cell lines NTERA2 (lane 2), MCF7 (lane 3), HeLa (lane 4), PC-3 (lane 5), PANC-1 (lane 6), and H1299 (lane 7) correspond to human TGCT cells, human mammary gland adenocarcinoma cells, human cervix adenocarcinoma cells, human prostate adenocarcinoma cells, human pancreas ductal epithelioid carcinoma cells, and human non-small cell lung cancer cells, respectively. α/β-Tubulin served as a loading control.

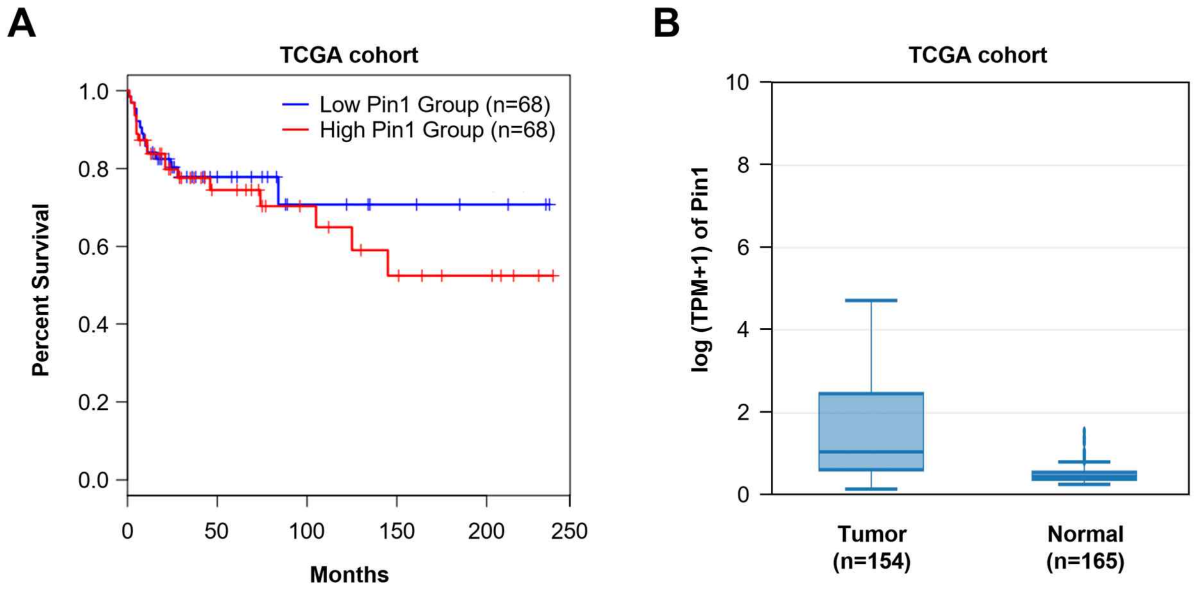

Supplementary Figure S3. Association of Pin1 expression with patients' survival. A. Disease-free survival curve for overall survival based on Pin1 expression in the TCGA cohort. To examine whether Pin1 plays an essential role in TGCTs, Kaplan–Meier survival curves for overall survival were generated based on Pin1 expression in the TCGA cohort (n = 68). **B. Graphical representation of differential expression of Pin1 in tumor (TGCT) and standard testis samples from TCGA.** Pin1 expression levels are shown in TGCT patients (Tumor, n=154) and testis tissue (Normal, n=165) in the TCGA cohort. TPM: transcripts per million. $p < 10^{-15}$.

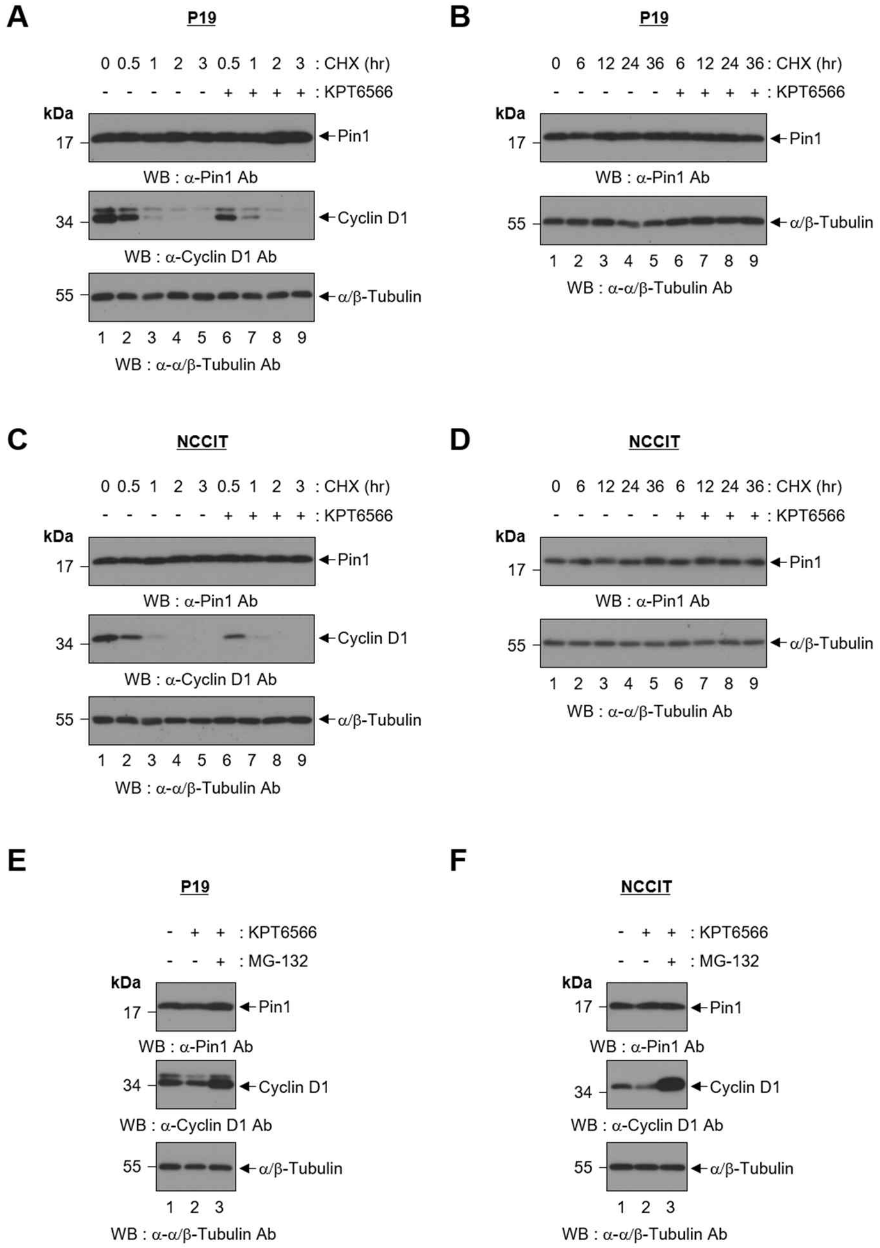

Supplementary Figure S4. No significant effect of KPT6566 on degradation of Pin1 in P19 and NCCIT cells. (A–D) Effect of KPT6566 and CHX on Pin1 protein levels in P19 and NCCIT cells. P19 (A and B) and NCCIT (C and D) cells were treated with 10 μg/mL CHX with (+) or without (-) 5 μM KPT6566 for the indicated durations (A and C, 0–3 hours; B and D, 0–36 hours). The Pin1 protein level was measured by western blot analysis. Cyclin D1 was used as a positive control (A and C, middle panels). α/β-Tubulin was used as a loading control and protein size markers are indicated on the left. KPT6566 treatment for up to 36 hours did not decrease the protein level of Pin1 in P19 (A and B) and NCCIT (C and D) cells. **(E–F) Levels of Pin1 in cells treated with KPT6566 and MG-132.** P19 (E) and NCCIT (F) cells were treated with 5 μM KPT6566 and 10 μM MG-132 for 16 hours. The levels of Pin1 and Cyclin D1 were measured by western blotting. Cyclin D1 was used as a positive control.

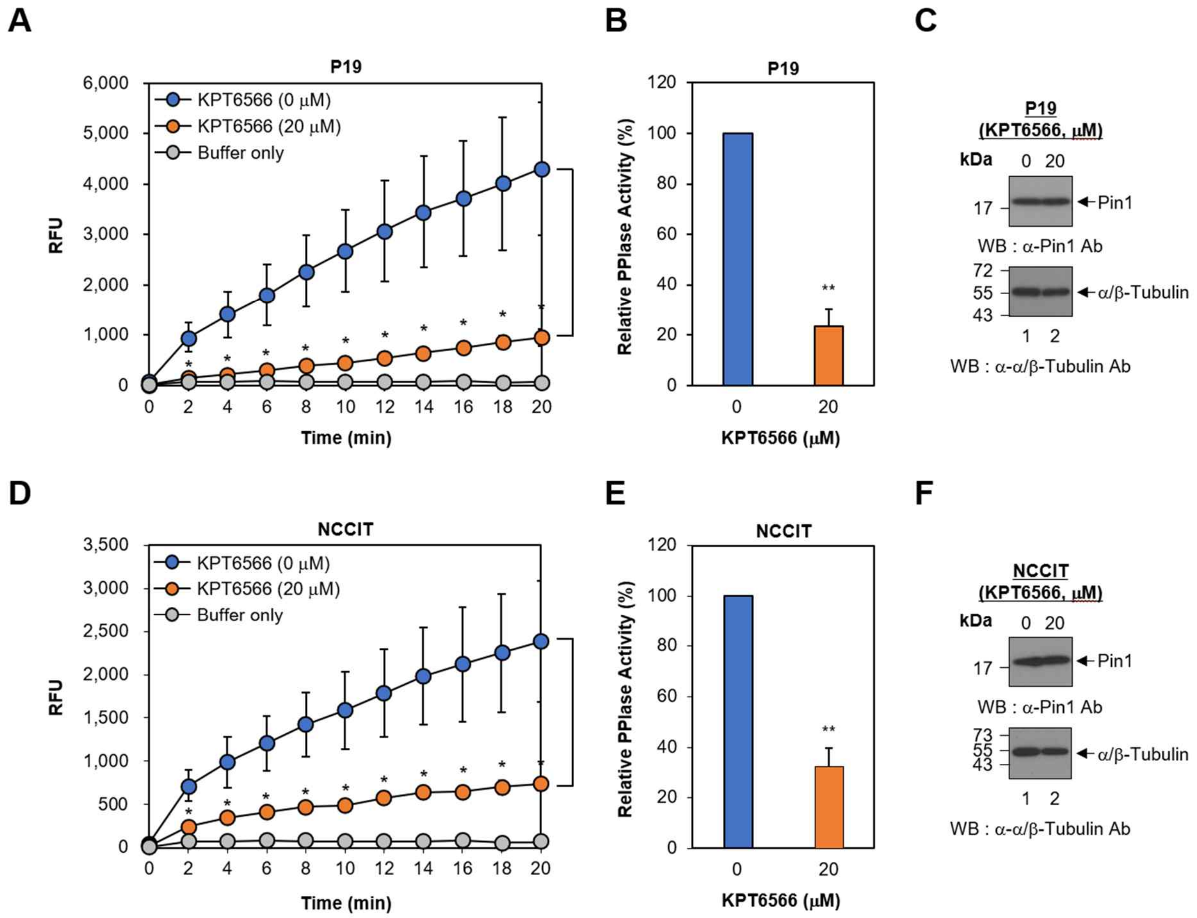

Supplementary Figure S5. Inhibition of the PPIase activity of Pin1 by KPT6566 in P19 and NCCIT cells. (A) Impact of KPT6566 on Pin1 PPIase activity in P19 cells. P19 cells were cultured with or without 20 μM KPT6566 for 48 hours. After preparation of total cell lysates, Pin1 activity was evaluated at 25°C using a SensoLyte® Green Pin1 Activity Assay Kit. The fluorescence intensity at excitation/emission = 490 nm/520 nm was monitored every 2 minutes for a total of 20 minutes. The presented values represent the mean ± S.D. (n=3; *$p < 0.05$ for 20 μM KPT6566 compared with control (0 μM KPT6566)). RFU denotes relative fluorescence units. **(B) Relative PPIase activity of Pin1 in P19 cells treated with KPT6566.** The PPIase activity of Pin1 in control (0 μM KPT6566) P19 cells was set to 100% for graphing purposes. n = 3. **$p < 0.01$ relative to control, unpaired Student's t-test. **(C) Immunoblot analysis of total P19 cell lysates used for the Pin1 activity assay.** Total P19 cell extracts used to evaluate Pin1 activity were subjected to western blotting. After being separated by SDS-PAGE, samples were probed with an anti-Pin1 or anti-α/β-Tubulin antibody. The molecular weights of the marker proteins (New England Biolabs) are displayed on the left (kDa). **(D) Effect of KPT6566 on Pin1 PPIase activity in NCCIT cells.** NCCIT cells were cultured in the absence or presence of 20 μM KPT6566 for 48 hours. Pin1 PPIase activity in total lysates of NCCIT cells was measured with a SensoLyte® Green Pin1 Activity Assay Kit. Data are mean ± S.D. (n=3; *$p < 0.05$ for KPT6566 compared with control (0 μM KPT6566)). **(E) Relative PPIase activity of Pin1 in NCCIT cells treated with KPT6566.** The PPIase activity of Pin1 in control (0 μM KPT6566) NCCIT cells was set to 100% for graphing purposes. n = 3. **$p < 0.01$ relative to control, unpaired Student's t-test. **(F) Immunoblot analysis of total NCCIT cell lysates used for the Pin1 PPIase activity assay.** Samples were separated by SDS-PAGE and then probed with an antibody against Pin1 or α/β-Tubulin. The molecular weights

of the marker proteins (New England Biolabs) are displayed on the left (kDa).

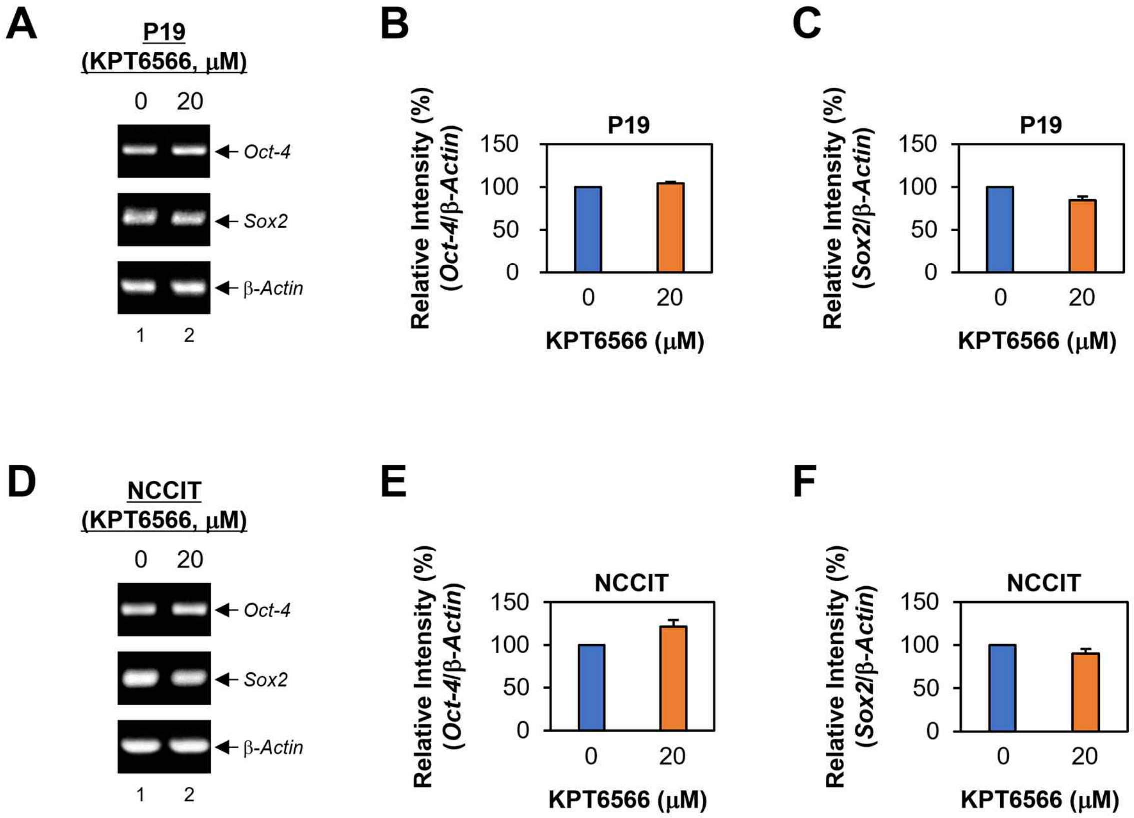

Supplementary Figure S6. Impact of KPT6566 on transcription of *Oct-4* and *Sox2*. (A and D) RT-PCR analysis of *Oct-4* and *Sox2* in P19 (A) and NCCIT (D) cells treated with KPT6566. After treatment with 20 μM KPT6566 for 48 hours, mRNA expression of *Oct-4* and *Sox2* in P19 (A) and NCCIT (D) cells was studied by RT-PCR. Subsequently, RT-PCR samples underwent agarose gel electrophoresis and ethidium bromide staining. β-*Actin* served as the reference gene. **(B, C, E, F) Graphical representation of *Oct-4* (B and E) and *Sox2* (C and F) mRNA expression in P19 (B and C) and NCCIT (E and F) cells treated with KPT6566.** Graphs display the mean ± S.D. of the relative levels of *Oct-4* (*Oct-4*/β-*Actin*) and *Sox2* (*Sox2*/β-*Actin*) from three independent experiments.
